# The Antibody Dependant Neurite Outgrowth Modulation Response Involvement in Spinal Cord Injury

**DOI:** 10.3389/fimmu.2022.882830

**Published:** 2022-06-16

**Authors:** Alice Capuz, Mélodie-Anne Karnoub, Sylvain Osien, Mélanie Rose, Céline Mériaux, Isabelle Fournier, David Devos, Fabien Vanden Abeele, Franck Rodet, Dasa Cizkova, Michel Salzet

**Affiliations:** ^1^ Université de Lille, Inserm U1192, Laboratoire Protéomique, Réponse Inflammatoire et Spectrométrie de Masse (PRISM), Lille, France; ^2^ Institut Universitaire de France, Paris, France; ^3^ Université de Lille, Inserm U1172, CHU-Lille, Lille Neuroscience Cognition Research Centre, Lille, France; ^4^ Université de Lille, Inserm U1003, Laboratory of Cell Physiology, Villeneuve d’Ascq, France; ^5^ Institute of Neuroimmunology, Slovak Academy of Sciences, Bratislava, Slovakia; ^6^ Centre for Experimental and Clinical Regenerative Medicine, University of Veterinary Medicine and Pharmacy in Kosice, Kosice, Slovakia

**Keywords:** spinal cord injury acute phase, inflammation, astrocytes, shot gun proteomic, neurite outgrowth, neuronal antibodies, autoantibodies, immunoglobulin

## Abstract

Spinal cord injury (SCI) represents a major medical challenge. At present, there is still no cure to treat it efficiently and enable functional recovery below the injury site. Previously, we demonstrated that inflammation determines the fate of the physiopathology. To decipher the molecular mechanisms involved in this process, we performed a meta-analysis of our spatio-temporal proteomic studies in the time course of SCI. This highlighted the presence of IgG isotypes in both spinal cord explants and their secretomes. These IgGs were detected in the spinal cord even if no SCI occurred. However, during the time course following SCI, abundance of IgG1 and IgG2 subclasses (a, b, c) varied according to the spatial repartition. IgG1 was clearly mostly abundant at 12 h, and a switch to IgG2a was observed after 24 h. This IgG stayed predominant 3, 7, and 10 days after SCI. A protein related to IgM as well as a variable heavy chain were only detected 12 h after lesion. Interestingly, treatment with RhoA inhibitor influenced the abundance of the various IgG isotypes and a preferential switch to IgG2c was observed. By data reuse of rat dorsal root ganglion (DRG) neurons RNAseq datasets and RT-PCR experiments performed on cDNA from DRG sensory neurons ND7/23 and N27 dopaminergic neural cell lines, we confirmed expression of immunoglobulin heavy and light chains (constant and variable) encoding genes in neurons. We then identified CD16 and CD32b as their specific receptors in sensory neuron cell line ND7/23 and their activation regulated neurites outgrowth. These results suggest that during SCI, neuronal IgG isotypes are released to modulate neurites outgrowth. Therefore, we propose a new view of the SCI response involving an antibody dependent neurite outgrowth modulation (ADNM) which could be a precursor to the neuroinflammatory response in pathological conditions.

## Introduction

The spinal cord and the brain form the central nervous system (CNS). It supports all nerves, coming from or going to the peripheral nervous system. It is protected by the spinal canal, formed by the articulation of cervical, thoracic, and lumbar vertebrae. This is why vertebral fracture or luxation can lead to spinal cord injury (SCI), from concussion/contusion to section (1). The clinical consequences of these lesions are terrible, as they lead, according to the level, either to paraplegia or tetraplegia, which can be complete or incomplete. According to the literature, the incidence ranges between 19.4 patients per year in Europe, and 51 patients per year in North America (2). This represents not only an epidemiologic issue, but also a social and economic burden, since the population affected is mostly represented by young men with a mean age of 40 years old. The SCI etiologies gather car crashes (31.47%), falls (25.29%), gunshot wounds (10.42%), motorcycle crashes (6.80%), and diving (4.67%) (2).

Although a fundamental understanding has been obtained through various modeling studies, there is still a lack of knowledge about the pathophysiology of SCI. It has been established that everything starts with a primary mechanic traumatism by compression, laceration, distraction, or shearing (3). This induces vascular lesions that lead to the formation of a hematoma at the lesion site, which itself causes local ischemia, edema, and cell-regulated cell death such as apoptosis (3). Moreover, the blood spinal cord barrier is disrupted, which initiates the secondary extension of the lesion through the attraction of inflammatory cells, the accumulation of cytokines and vasoactive proteins. Finally, cell death is responsible for the accumulation of cellular debris, potassium, and ATP, leading to a cytotoxic environment. Then pro-inflammatory M1 microglial cells and phagocytes infiltrate this cytotoxic environment resulting in toxic oxidative stress to adjacent neurons (3). In our laboratory, a previous study focused on subacute inflammatory response after SCI demonstrated that after 3 days, the inflammatory response was intense at the lesion site and in downstream caudal 1 segment. Indeed, high amounts of cytokines and immunoglobulins were detected and the Rho-Rock and Memo1-RhoA-Diaph1 pathways were activated (4, 5). On the contrary, more caudally and distally from the central lesion, we have rather detected neurite outgrowth proteins and chemokines attracting first neutrophils, then T regulatory cells, and anti-inflammatory M2 microglial cells. This created a favorable environment for neurite outgrowth on both sides of the lesion (4).

Several innovative treatments currently being tested inhibit ligands known to engage the Rho-Rock pathway in neurons. These notably include chondroitin sulfate proteoglycans, myelin associated glycoprotein (MAG), oligodendrocyte myelin glycoprotein (OMGP), and neurite outgrowth inhibitor A (NOGO-A). For example, anti-NOGO-A antibodies delivered in the cerebrospinal fluid have been used in phase I clinical trials (6, 7) with promising results. Also, in pre-clinical studies, chondroitinase ABC was shown to partially block the Rho-Rock-mediated inhibition of neurite outgrowth and glial scar formation within the lesion (8). Finally, a RhoA inhibitor (RhoAi), called Cethrin (9, 10), has been assessed in a phase I and phase IIa clinical trial in patients with cervical SCI, and was found to improve functional repair (11). A phase IIb is foreseen. However, we have recently shown that the mechanisms triggered 12 h after the lesion were completely different from those observed after 1 day of injury. At 12 h after SCI, rostral and caudal segments showed no molecular difference but were distinct from the injury site (12). Repetitive delivery of RhoA inhibitor significantly increased the synaptogenesis and promoted neuritogenesis and neurite outgrowth through the cavity (12). However, a week after lesion, a plateau appeared which blocked the achievement of the biological processes and the neuronal reconnection. Among the proteins that may be involved in this inhibition, IgG1 and IgG2 isotypes represent good candidates. Indeed, we observed their presence in SCI secretome as early as 12 h after the lesion (4, 12). Then, we confirmed their presence in neurons and astrocytes 24 h after SCI even after preventive treatment with anti-CD20 (4, 12). In this context, we decided to identify these immunoglobulins between 12 h and 7 days after SCI. A meta-analysis was performed with MaxQuant software, in two rows. First analysis was performed against the SCI data (4) at several times (12 h, 1 day, 3 days, 7 days, and 10 days), and with several segments including rostral (R3, R2, R1), lesion (L), and caudal (C1, C2, C3). We then studied their expression and that of their specific receptors in neurons as well as their activity related to neurite outgrowth.

## Materials and Methods

### Reagents

All chemicals were obtained with the highest purity available. Water, acetonitrile (ACN), formic acid (FA), and trifluoroacetic acid (TFA) were purchased from Biosolve B.V. (Valkenswaard, the Netherlands). DL-dithiothreitol (DTT), HEPES, ES FBS, thiourea, iodoacetamide (IAA), tri-reagent, isopropanol, chloroform, glucose, and agarose were purchased from Sigma Aldrich. Lys-C/Trypsin enzymatic mixture, DNase RQ1, deoxyribonucleotides (dNTPs), RNAsin^®^ ribonuclease inhibitor, RNase H, GoTaq^®^ G2 Hot Start Taq polymerase kit, molecular weight markers, PGEM-T Easy Vector System II^®^, T4 DNA ligase, and *E. coli* strain JM109 were purchased from Promega (France). Random primers, Superscript^®^ III kit, phosphate buffered saline (PBS), Dulbecco’s modified Eagle’s medium (DMEM), RPMI 16140, fetal bovine serum (FBS), L-glutamine, penicillin and streptomycin, GeneJET Gel Extraction kit, Alexa Fluor^®^ 647-conjugated goat anti-rat IgG, Alexa Fluor^®^ 488-conjugated donkey anti-rabbit, Alexa Fluor^®^ 488-conjugated donkey anti-mouse, melon Gel IgG Spin Purification Kit and Zeba Spin Desalting Columns (7K MWCO), Dynabeads Protein G, and enhanced chemiluminescence kit were obtained from Life Technologies (Milan, Italy). RhoA inhibitor was obtained from Cytoskeleton, Inc. (Denver, CO). All primers used were purchased from Eurogentec. The QiAquick PCR Purification kit was purchased from Qiagen^®^. The NucleoSpin^®^ Plasmid kit was purchased from Macherey Nagel. Urea was purchased from Euromedex. Mouse anti-GFAP, mouse anti-NeuN, Amicon ultracentrifugal filter 10K, and ZipTip C18 were purchased from Millipore. Mouse anti-CD16 and rabbit anti-CD32 were obtained from Santa Cruz Biotechnology. Peroxidase-conjugated goat anti-rat IgG was obtained from Thermofisher scientific, peroxidase-conjugated goat anti-mouse IgG and peroxidase-conjugated goat anti-rabbit IgG were obtained from Jackson ImmunoResearch (West Grove, PA). Ultrapure lipopolysaccharides (LPS-EB) were bought from *In vivo*Gen (Toulouse, France). PNGase F was bought from New England’s Biolabs (US).

### Experimental Design and Statistical Rational

Spinal cord segments and their conditioned media during time course of experiments were collected from at least 3 rats. For the proteomic statistical analysis of conditioned media, as a criterion of significance, we applied an ANOVA significance threshold of P<0.05, and heat maps were generated. Normalization was achieved using a Z-score with a matrix access by rows. Western blot analyses were performed twice in duplicate. Other experiments such as molecular biology, neurites outgrowth, and calcium measurements were conducted in triplicate. For neurite outgrowth experiments, statistical significance was assessed with a t-test using Graph pad PRISM software. Values of P < 0.05 were considered statistically significant (*P value of < 0.05, **P value of < 0.01, ***P value of < 0.001).

### Secretome Preparation

All studies were carried out on adult male Wistar rats with the agreement and according to the rules laid down by the institutional committee for the protection of animals of the Slovak University of Science and by the European Directive 2010/63 on the use of the animals for research purposes, as well as with the Slovak animal welfare laws Nos. 377/2012 and 436/2012. Some of the animals underwent spinal cord injury at Th8-Th9 level by the balloon compression method, performed after laminectomy. Other animals, called “control” did not undergo compression but a simple laminectomy. Some also received treatment with RhoA inhibitor, injected at the lesion site at the time of the lesion. Rats were sacrificed after isoflurane anesthesia, 12 h (3 rats not treated with RhoA inhibitor and 3 rats treated with RhoA inhibitor) or 24 h (5 rats) after injury. Spinal cords were extracted by injection of sterile saline buffer and divided into 1-cm long segments, on both sides of the lesion, which level was macroscopically verified. It was therefore possible to obtain a lesion segment, 2 rostral segments, and 2 caudal segments, each segment being cut into two fragments of 0.5 cm. These fragments were then cultured for 12 h and 24 h in DMEM at 37°C and 5% CO_2_. Conditioned media thus obtained were called secretomes.

### Preparation of Intact Spinal Cord

Rats were sacrificed after isoflurane anesthesia. The spinal cord was extracted by injection of sterile saline buffer and immediately frozen as a whole.

### Cell Line

The rat dorsal root ganglion (DRG) cell line ND7/23 and N27 dopaminergic neural cell line were purchased from Sigma-Aldrich. ND7/23 cells were obtained by PEG mediated cell fusion between the mouse neuroblastoma (N18 tg 2) and the rat dorsal root ganglion neurons. ND7/23 cell line was grown in DMEM medium supplemented with 10% fetal bovine serum, 1% L-glutamate, 100 U/ml penicillin, and 100 μg/ml streptomycin, at 37°C in a humidified atmosphere (5% CO_2_). N27 cells were cultivated in RPMI 1610 supplemented with 10% embryonic FBS, 1% L-glutamate, 100 U/ml penicillin, and 100 μg/ml streptomycin, at 37°C in a humidified atmosphere (5% CO_2_).

### Protein Extraction

To extract the proteins, ND7/23 DRG cells were resuspended in RIPA buffer (150 mM NaCl, 50 mM Tris, 5 mM EGTA, 2 mM EDTA, 100 mM NaF, 10 mM sodium pyrophosphate, 1% Nonidet P-40, 1 mM PMSF, and 1X protease inhibitors) and subjected to three sonications of 5 s with a step on ice for 30 s between each sonication. Then the samples were centrifuged at 14,000 x g for 20 min at 4°C. The supernatant containing the proteins was collected. To determine protein concentration in the samples, Bradford assay was used.

As a first step to purify antibodies from spinal cord lesion segment withdrawn 7 days after injury (SCI 7D-L), proteins were extracted from 50 mg of tissue resuspended in RIPA buffer and submitted to the same procedure as described above. Seven days were chosen to mimic the dynamic of the inflammatory response that occured 7 days after lesion.

To conduct shotgun proteomics analysis on non-injured spinal cords, control spinal cords as well as on rostral, lesion, and caudal spinal cord segments collected 12 h after SCI, protein extraction was performed as follows. Tissues were cut to obtain small fragments of 1 mm thick and grinded in liquid nitrogen. Powders were resuspended in CHAPS lysis buffer (CHAPS 3.5%, Tris-HCl and DTT, pH 10), mixed thoroughly and subjected to sonication during 20 min. Samples were heated at 95°C during 5 min and centrifuged at 15,000 x g during 5 min at 4°C. Supernatants containing proteins were then collected.

### Immunoglobulin Enrichment Procedure From SCI 7D-L Protein Extracts

To purify antibodies from protein extracts of SCI 7D-L obtained after RIPA extraction, ammonium sulfate precipitation was carried out on 500 µg of proteins according to the protocol of the Melon Gel IgG Purification Kit, with a few modifications. Briefly, saturated ammonium sulfate solution was prepared by dissolving 7.61 g ammonium sulfate in 10 mL milliQ water. Then, one volume of saturated ammonium sulfate solution was added to one volume of sample in order to have 50% ammonium sulfate. The sample was incubated during 4 h at 4°C, and centrifuged at 3000 x g for 20 min at 4°C. The supernatant was then removed and the pellet was resuspended in a volume of milliQ water, equivalent to the original sample volume. The resuspended pellet containing the purified antibodies was desalted thanks to the Zeba Spin Desalting Columns according to manufacturer’s instructions. Column’s caps were removed and the column was placed in a 2.0-mL collection tube, prior to centrifugation at 1500 x g for 1 min in order to remove the storage solution. A washing step was performed by adding 300 µL of milliQ water to the column, and centrifugation at 1500 x g for 1 min. The washing process was repeated two more times. Ensuing these steps, sample was carefully added to the center of the column, and centrifuged at 1500 x g for 2 min in order to collect the desalted sample. Melon Gel IgG Purification was then conducted according to the manufacturer’s instructions. Purification Buffer and Melon Gel IgG Purification Support were equilibrated for 15 min at room temperature. During this time, the sample volume was completed to 500 µL with purification buffer. Then, 500 µL of slurry was dispensed into a spin column placed in a microcentrifuge tube, and centrifuged for 1 min at 4000 x g. The flow-through was discarded afterward, and two washes were performed by adding 300 µL of purification buffer and centrifugation at 4000 x g for 10 s. The flow-through was discarded again, and the sample was loaded into the column. The column was mixed end-over-end for 5 min at room temperature, in order to bind the nonspecific proteins to the resin. The bottom cap was removed, the top cap was loosed, and centrifugation was performed at 4000 x g for 1 min to collect the purified antibodies in a new collection tube. After melon gel purification, the sample was digested after reduction, alkylation, and deglycosylation.

### Reduction, Alkylation, Deglycosylation, and Trypsic Digestion of Samples Obtained After Melon Gel IgG Purification

Reduction was performed by the addition of DTT 200 mM to the sample, in order to have a final concentration of 50 mM, and the sample was incubated at 56°C for 1 h. Then, the sample was loaded into an Amicon 10K to change the buffer and retain the proteins on the membrane. To do that, approximately 450 µL was loaded into the column, centrifugation at 14,000 x g was done for 15 min, and flow-through was discarded. This step was performed four times in order to load the entire sample, since the maximum capacity of the Amicon was 500 µL. Then the washes step was performed by the addition of 200 µL NH_4_HCO_3_ 50 mM to the column, centrifugation at 14,000 x g for 15 min, and flow-through removal. Alkylation was then performed by the addition of 100 µL IAA 50 mM, mixed briefly at 600 RPM, and incubated in the dark for 20 min. Then centrifugation was performed at 14,000 x g for 15 min, and two washes were performed by the addition of 200 µL NH_4_HCO_3_ 50 mM, centrifugation at 14,000 x g for 15 min, and flow-through removal. Ensuing these steps, deglycosylation with PNGase F was carried out. PNGase F was prepared with NH_4_HCO_3_ 50 mM (1:99, v/v), and a volume of this solution was added to the sample in order to have 10% of diluted PNGase F in the final sample. Then, the sample was mixed briefly at 600 RPM and incubated overnight at 37°C. The next day, two washes were performed by the addition of 200 µL NH_4_HCO_3_ 50 mM to the column, centrifugation at 14,000 x g for 15 min, and flow-through removal. After the addition of 40 µL of Trypsin (20 ng/µL in NH_4_HCO_3_ 50 mM), the sample was mixed briefly at 600 RPM and incubated overnight at 37°C. The addition of trypsin quenched the reaction with PNGase F. The next day, the column was placed in a new collection tube and 40 µL NH_4_HCO_3_ 50 mM were added. A centrifugation was performed at 14,000 x g for 10 min to collect the digested sample. Trypsin digestion was then quenched with TFA 1%. Finally, the sample was dried in the SpeedVac and desalted with ZipTip C18 before HPLC-MS/MS analysis.

### Reduction, Alkylation, and Trypsic Digestion of Secretomes From Spinal Cord Segments Treated Or Not With RhoA Inhibitor Collected 12 h and 24 h After SCI

Secretomes were deposited on 0.22-µm syringe filters. After filtration, denaturation of the proteins contained in the secretomes was performed with Urea 6 M and HEPES 40 mM. Reduction was then carried out with DTT 10 mM at 56°C for 40 min. Afterward, alkylation was conducted with IAA 55 mM for 40 min at room temperature and obscurity. Thiourea 100 mM was added to stop the reaction and digestion was performed overnight at 37°C with Lys-C/Trypsine (30 μg/mL). Addition of TFA 0.5% stopped the reaction. Finally, samples were dried in the SpeedVac and desalted with ZipTip C18 before HPLC-MS/MS analysis.

### Reduction, Alkylation, and Trypsic Digestion of Protein Extracts From Non-Injured Spinal Cords, Control Spinal Cords As Well As Rostral, Lesion, and Caudal Spinal Cord Segments Collected 12 h After SCI

A total of 30 μl of each sample was loaded into an Amicon 30K and 200 µL of UA buffer (Urea 8 M, Tris-HCl 0.1 M pH 8.5) were added. Samples were centrifuged at 14,000 x g for 15 min and flow-through was discarded. Alkylation was conducted with 100 µL of IAA 55 mM. UA buffer was then added and samples were centrifuged at 14,000 x g for 15 min. This step was repeated three times. Ammonium bicarbonate (AB) buffer was then added and samples were centrifuged three times at 14,000 x g for 15 min. Digestion was performed overnight at 37°C with Lys-C/Trypsine (30 μg/mL). After centrifugation at 14,000 x g for 10 min, 500 µL of NaCl and TFA 0.5% were added. Finally, samples were dried in the SpeedVac and desalted with ZipTip C18 before HPLC-MS/MS analysis.

### SDS-PAGE and In Gel Digestion

To identify immunoglobulins in secretomes of segments R1, L, and C1 collected 12 h and 24 h post SCI, 2.5 μg of proteins of each sample were separated on a 12% SDS-polyacrylamide gel (SDS-PAGE). Gels were colored overnight at room temperature with Coomassie blue and in gel digestion was eventually carried out. Bands of interest were cut in small pieces of approximately 1 mm square and placed in new microtubes. Then 300 µl of milliQ water were added to the gel pieces and mixed for 15 min. Then 300 µL of ACN were added and the samples were mixed for 15 min. The supernatant was removed afterward, and 300 µL of NH_4_HCO_3_ 100 mM were added to the gel pieces. After an agitation of 15 min, supernatants were removed. A total of 300 μl of ACN/NH_4_HCO_3_ 100 mM (1/1, v/v) were added. After an agitation of 15 min, supernatants were discarded. As a final wash, 100 µL ACN were added to the gel pieces and an agitation of 10 min was carried out. Band pieces should become white and shrinked. Then ACN was removed and band pieces were dried in the SpeedVac for 10 min. Reduction was performed on the band pieces by the addition of 50 µL/band DTT 10 mM in NH_4_HCO_3_ 100 mM, and incubation at 56°C for 1 h. Following this step, 50 µL/band IAA 50 mM in NH_4_HCO_3_ 100 mM were added to the gel pieces and samples were incubated for 30 min in the dark at room temperature. Then supernatants were removed and 300 µL NH_4_HCO_3_ 100 mM were added and mixed for 15 min. Supernatants were removed again, and 300 µL ACN/NH_4_HCO_3_ 20 mM (1/1, v/v) were added to the band pieces and mixed for 15 min. Ensuing this agitation, supernatants were removed and a final wash with 100 µL ACN was performed with an agitation during 10 min. Band pieces should become white and shrinked. ACN was removed from the samples and band pieces were dried for 10 minutes in the SpeedVac. Digestion was then performed. Then 50 µl of Trypsin (20 ng/µL) were added to the band pieces, and incubation at 37°C was performed overnight. The next day, after an addition of 50 µL of ACN, samples were incubated at 37°C for 30 min and mixed for 20 min. The supernatants containing the peptides were transferred afterward to new microtubes. After an addition of 50 µL of 1% FA, band pieces were mixed for 20 min, and the supernatants were collected in the new microtubes from the previous step. This last step was carried out four times in total. Afterward, 150 µL ACN were added to the band pieces, followed by an agitation during 10 min. The supernatants were collected and added to the tubes containing the peptides. The peptides were finally dried in the SpeedVac and desalted with ZipTip C18 prior to HPLC-MS/MS analysis.

### HPLC-MS/MS

An online reversed-phase chromatography was used to separate the sample, through a Thermo Scientific Proxeon easy nLC1000 equipped with a Proxeon trap column (100 μm ID x 2 cm, Thermo Scientific) and a C18 packed-tip column (Acclaim PepMap, 75 µm ID x 15 cm, Thermo Scientific). Peptides were separated with an increasing amount of ACN (2%-40% over 60 min) at a flow rate of 300 nL/min. The peptides were electrosprayed directly from the analytical column and a voltage of 1.7 kV was applied *via* the liquid junction of the nanospray source. The chromatography system was coupled to the mass spectrometer Thermo Scientific Q-exactive programmed with a top 10 data-dependent mode for all the samples. The resolving power was 70,000 FWHM (m/z 400), in a positive mode and using an AGC target of 3^e6^. Default charge state was set at 2, unassigned and +1 charge states were rejected and dynamic exclusion was enabled for 25 s. The scan range was set to 300-1600 m/z. For ddMS², the scan range was between 200 and 2000 m/z, 1 microscan was acquired at 17,500 FWHM and an isolation window of 4.0 m/z was used.

### MS Data Analysis

The MS data from secretomes were analyzed through MaxQuant 1.6.2.6 using the Andromeda search engine. Proteins and peptides were searched against a custom databank composed of the complete proteome of Rattus norvegicus (29,961 entries, July 2018) from the Uniprot database. Carbamidomethylation was set as a static modification, and methionine oxidation was set as a variable modification. For samples subjected to the deglycosylation step, asparagine to aspartate was also set as a variable modification. Parameters were set to 1 peptide per protein, 2 miss cleavages, with a strict FDR of 0.01. The mass tolerance was 10 ppm for the precursors, and 0.6 ppm for the fragments. Relative, label-free quantification was performed using the MaxLFQ algorithm integrated in MaxQuant software, with the default parameters. LFQ intensity was logarithmized (log2[x]). Proteins only identified with modified peptides and potential contaminants were removed. The MS data from neuron samples were analyzed through Proteome Discoverer 2.2. Proteins and peptides were searched against the same custom databank as for the secretome samples, in order to identify the VHH. Carbamidomethylation was set as a static modification, and methionine oxidation was set as a variable modification. For samples subjected to the deglycosylation step, asparagine to aspartate was also set as a variable modification. Parameters were set to 1 peptide per protein, 3 miss cleavage, with no limitation for FDR to identify all the potential peptides in the sample. Mass tolerance was also 10 ppm for the precursors and 0.6 ppm for the fragments.

### Alignments of Peptidic Sequences

For the alignments of the variable parts of the antibodies, and for some of the constant parts of the antibodies, peptides were identified in different entries of the protein databank because of a high variability in the peptidic sequences. To align all the peptides obtained by MS, entries of the same part of the antibodies were aligned first in MultiAlin (http://multalin.toulouse.inra.fr/multalin/), then the consensus was used to perform the alignments. All the peptides identified were directly highlighted on the consensus sequence to compare peptides identified in all the samples.

### Western Blots

To detect the presence of immunoglobulins in secretomes of segments R1, L, and C1 collected 12 h and 24 h after SCI, Western blots were performed (n=2). To determine protein concentration in the samples, the Bradford method was used. Twelve percent acrylamide gels were loaded with 2.5 μg of proteins from each sample. After migration, proteins were transferred to a nitrocellulose membrane. Membranes were saturated for 1 h in a solution of PBS-Tween 0.1% containing 5% non-fat dry milk and incubated overnight at 4°C with peroxidase-conjugated goat anti-immunoglobulins (0.08 µg/mL). After intensive washes with PBS-Tween 0.1%, chemiluminescence revelation was performed. Membranes were then dehybridized for 30 min with 0.2 M citric acid solution. After intensive washes with PBS-Tween 0.1%, membranes were again saturated and incubated overnight at 4°C with a primary mouse anti-GFAP antibody (1:1000). After intensive washes with PBS-Tween 0.1%, membranes were incubated with a peroxidase-conjugated goat anti-mouse antibody (0.03 µg/mL). After intensive washes with PBS-Tween 0.1%, chemiluminescence revelation was performed.

To detect the presence of immunoglobulins in secretomes of segments R1, L, and C1 from rat treated with RhoA inhibitor, injected at the lesion site at the time of the lesion and collected 12 h after SCI, Western blots were performed (n=2). Secretomes of segments R1, L, and C1 from rat not treated with RhoA inhibitor and collected 12 h after SCI served as controls. To determine protein concentration in the samples, the Bradford method was used. Twelve percent acrylamide gels were loaded with 2.5 μg of proteins of 12 h samples. After migration, proteins were transferred to a nitrocellulose membrane. Membranes were saturated for 1 h in a solution of PBS-Tween 0.1% containing 5% non-fat dry milk and incubated overnight at 4°C with peroxidase-conjugated goat anti-immunoglobulins (0.08 µg/mL). After intensive washes with PBS-Tween 0.1%, chemiluminescence revelation was performed.

To detect FC gamma receptor CD16 in DRG ND7/23 cells, an immunoprecipitation was carried out. Anti-CD16 diluted at 1:100 in PBS-Tween 0.02% was added to Dynabeads Protein G and incubated for 4 h. Afterward, 1 mg of protein extracts from ND7/23 cells treated or not with 200 ng/mL of LPS was added and incubated overnight at 4°C. After intensive washes with PBS-Tween 0.02%, Protein G beads were eluted with glycine 50 mM pH 2.8 and Tris buffer pH 7.4 was added to neutralize the pH. Proteins eluted were separated by SDS-PAGE and transferred onto nitrocellulose membranes. After saturation for 1 h with the blocking buffer (PBS-Tween 0.1% containing 5% BSA), the membranes were incubated overnight at 4°C with mouse anti-CD16 (1:500) diluted in the blocking buffer. After intensive washes with PBS-Tween 0.1%, membranes were incubated with peroxidase-conjugated goat anti-mouse (0.03 µg/mL). After intensive washes with PBS-Tween 0.1%, chemiluminescence revelation was performed.

To detect FC gamma receptor CD32 in ND7/23 DRG cells, 40 µg of total cell extracts were analyzed by Western blotting. Proteins were separated by SDS-PAGE and transferred onto nitrocellulose membranes. After saturation for 1 h with the blocking buffer (PBS-Tween 0.1% containing 5% BSA), the membranes were incubated overnight at 4°C with rabbit anti-CD32 (1:500) diluted in blocking buffer. After intensive washes with PBS-Tween 0.1%, membranes were incubated with peroxidase-conjugated goat anti-rabbit (0.08 µg/mL). After intensive washes with PBS-Tween 0.1%, chemiluminescence revelation was performed.

### Spinal Cords Sections and Immunofluorescence

Spinal cords were collected and fixed in 4% paraformaldehyde. They were then submerged in sucrose baths of increasing concentration of 10 to 30% over 3 days before being included in 2% cellulose and frozen at -80°C. Samples were then cut with a cryostat in 20-μm thick sections. For IHC, sections were dried in a desiccator for 5 min, followed by antigen retrieval in Tris-HCl 20 mM pH 9 by 30 s microwaves treatment. Sections were then rehydrated by 3 baths of phosphate buffer (PBS 1X), before saturation in PBS 1X containing 1% BSA buffer, 1% ovalbumin, 0.05% triton, and 1% normal donkey serum. Alexa Fluor^®^ 647-conjugated goat anti-rat IgG (4µg/mL) and mouse anti-NeuN (1:500) were then added followed by an overnight incubation at 4°C. Sections were then washed 3 times with PBS 1X, before incubation for 1 h at 37°C with Alexa Fluor^®^ 488-conjugated donkey anti-mouse IgG (2 µg/mL). After intensive washes with PBS 1X, coverslips were mounted using Dako fluorescent mounting medium.

### CD16 and CD32b Immunostaining and Fluorescence Quantification

To detect Fc gamma receptors, 35,000 ND7/23 cells were grown on coverslips and treated or not beforehand with LPS (200 ng/ml) and RhoA inhibitor (1 µg/mL) for 24 h. The cells were fixed with paraformaldehyde 4% (PAF) for 10 min, washed in PBS1X (phosphate buffered sodium 1X), and quenched with glycine 50 mM. After cell membrane permeabilization with 0.2% Triton X-100 for 10 min, cells were immersed in a blocking buffer (PBS 1x containing 1% bovine serum albumin, 1% ovalbumin, 1% NDS) for 1 h. The cells were then incubated overnight at 4°C with rabbit anti-CD32 (1:100) or mouse anti-CD16 (1:100) diluted in the blocking buffer. Washes with PBS 1x were performed and followed by 1 h incubation at 37°C with Alexa Fluor^®^ 488-conjugated donkey anti-rabbit or Alexa Fluor^®^ 488-conjugated donkey anti-mouse diluted at 2 µg/mL in the blocking buffer. The cells were further washed with PBS 1X and nuclei were stained with Hoechst 33,342 (1:10 000). After a final wash in PBS 1X, coverslips were mounted using Dako fluorescent mounting medium. Fluorescent stained cells were analyzed using a confocal microscope (Zeiss LSM700) and the quantification was performed by ImageJ software. To that end, 10 fields representative of the wells were selected for each condition and circularity, area, and mean fluorescence as well as the background of each cell presented in the field were measured. Fluorescence intensity was calculated with the total corrected cellular fluorescence (TCCF) equation = integrated density – (area of selected cell × mean fluorescence of background readings). The results are presented as means ± SD. The statistical significance was evaluated through Student’s t-test and values of p < 0.05 were considered statistically significant (*p-value of <0.05).

### Investigation of the Biological Effects of Fc Gamma Receptors in ND7/23 DRG Cells

To study the role of CD16 and CD32 receptors, ND7/23 DRG cells were placed in serum-free medium containing 1% L-glutamate, 100 U/ml penicillin, and 100 μg/ml streptomycin. Afterward, cells were treated for 1 h with 1 µg/mL of RhoA inhibitor and then incubated for 24 h with rabbit anti-CD32 (1:600), mouse anti-CD16 (1:100), and mouse anti-GFAP (1:500). Mouse anti-GFAP displayed an IgG2 isotype and served as a control of Fc gamma receptors activation. Following these treatments, neurites length was measured through Image J software and the statistical significance was evaluated with a t-test using Graph pad PRISM software. Values of P < 0.05 were considered statistically significant (*P value of < 0.05, **P value of < 0.01, ***P value of < 0.001).

### Calcium Homeostasis Analysis in ND7/23 DRG Cells

ND7/23 DRG cells were treated with rabbit anti-CD32 (1:500) and mouse anti-CD16 (1:500) for 24 h and loaded with 2.5 µM Fura-2 AM. After intensive washes with culture medium, fluorescence intensity of Fura-2 in cells was recorded at 340 and 380 nm using MetaFluor Software. The ratio F340/F380 was calculated to evaluate the variations in cytosolic Ca^2+^ concentrations. After 100 s of monitoring, 1 µM of thapsigargin was added to induce Ca^2+^ efflux from the ER. This led to the activation of store-operated channels (SOCs) located at the plasma membrane increasing Ca^2+^ intake into the cells.

### Transcriptomic Studies

To study *Fcgr3* (*CD16)* expression in ND7/23 DRG, RNA were extracted from ND7/23 cells stimulated with 200 ng/mL of LPS using Tri Reagent according to the manufacturer’s instructions. Two micrograms of RNA were treated with 2 units of DNase RQ1 and retro-transcribed using the SuperScript^®^ III kit. To perform RT-PCR experiments, the following primers were used: Rat *Fcgr3* (forward primer: 5′- CAC AGT CAA TGA CAG TGG -3′; reverse primer: 5′- TTG GAC ACA TGC ATT GTC -3′). cDNA amplification was performed using GoTaq polymerase and 40 cycles at 95°C/30 s, 60°C/1 min, and 72°C/1 min. Amplicons were then purified, subcloned into pGEM-T easy vector, and sequenced.

To study Rat *IgG2c* gene expression in N27 and ND7/23 cell lines, RNA were extracted from ND7/23 or N27 cells using Tri Reagent according to the manufacturer’s instructions. Two micrograms of RNA were treated with 2 units of DNase RQ1 and retro-transcribed using the SuperScript^®^ III kit. To perform RT-PCR experiments, the following primers were used: Rat *IgG2C* (forward primer: 5′- TCC GTG AAG CTC TCT TGT GC-3′; reverse primer: 5′- ATG GAG GCC TGG GAG GGA CGG -3′).

To study *Rag1* and *Rag2* gene expression in DRG cells (ND7/23) and N27 cell line, cells were stimulated 24 h with 10^-4^ M of H_2_O_2_. DRG cells were also stimulated with secretome from lesion segment collected 1 day after SCI. RNA were extracted using Tri Reagent according to the manufacturer’s instructions. Two micrograms of RNA were treated with 2 units of DNase RQ1 and retro-transcribed using the SuperScript^®^ III kit. To perform RT-PCR experiments, the following primers were used: Rat *Rag1* (forward primer: 5′- GGC CAT CCG TGT CAA TAC CT-3’; reverse primer: 5′- ACC GAA CTG CCT TTT CTG GA-3′), *Rag2* (forward primer: 5′- GCC TTC TAC CCA AAG AAC CAC-3’; reverse primer: 5′- ACA GTC CCG TTT CCC ATG TT-3′), *Actin* served as referent gene (forward primer: 5′- TTG TAA CCA ACT GGG ACG ATA TGG-3’; reverse primer: 5′- GAT CTT GAT CTT CAT GGT GCT AGG-3′).

### RNASeq Data Reuse Analyses

RNAseq libraries were obtained from the Sequence Read Archive (SRA) database. SRX11310972 corresponded to the dorsal root ganglion (DRG) explant. By contrast SRX7119488 and SRX10720738 were obtained from primary neurons from DRG. A blast search was carried out on each databank to identify CD markers and immunoglobulin heavy and light chains. Initially, the search was carried out with a maximum of 100 reads for the expressed genes and a second analysis was carried out with 500 reads. The alignment allowed the identification of reads at the 5’ end. Those reads covered the complete constant chain as well as the V(D)J region. This was confirmed using the IgBlast tool. The reads encoding a variable part linked to a constant part were aligned with a kappa light chain constant part.

## Results

In a past study proteins involved in the Memo-RhoA-Diaph1 and Rho-Rock pathways were identified in both lesion and caudal 1 segment on Day 3 to 10 post-SCI. On the opposite, rostral 1 segment was characterized by a pro-regenerative inflammatory profile favoring the attraction of neutrophils and T regulatory cells as well as a polarization of macrophages/microglia toward a repair promoting M2 phenotype (12). Accordingly, in these segments, many proteins promoting neurogenesis (neurotrimin, neurofascin, semaphorins) and synaptogenesis (septins, syntaxins, synapsins) were characterized. Such proteomic profiles contributed to settle a pro-neurite outgrowth medium on both parts of the lesion (12). Unfortunately, these phenomena were not only asynchronous but hampered by the development of a repair-inhibiting process that took place within the lesion itself and the adjacent caudal segment (thereafter referred to as the C1 segment). In these segments, activation of the Rho-Rock pathway, pro-inflammatory signals, glial scar-promoting molecules prevented the neurite outgrowth process initiated both in the adjacent rostral segment and the distal caudal one. Our objective was therefore to provide a precise description of time- and segment-specific inflammatory events and to identify new immune-related therapeutic targets in SCI. To prove that our model was well suited to assess the impact of inflammation during SCI, we first performed *in vivo* experiments using the anti-inflammatory agent, FK506 (12). Results showed that FK506 slightly improved synaptogenesis and neuritogenesis as revealed by anti-synaphysin I and anti-GAP43 labelling and increased the BBB score from 2 to 8 ± 2 in 2 weeks compared to untreated SCI rats. These results reflected that inflammation modulation could improve the BBB score and synaptogenesis but not neurite outgrowth (12). Therefore, it was not sufficient to greatly improve the regeneration process. In these conditions, we decided to deeply analyze the events occurring at early stages of the lesion, that is, 12 h and 24 h after SCI.

### Spatio-Temporal Organization of Inflammatory Response and Neurogenesis at the Acute Stage of SCI

**Figure 1:**
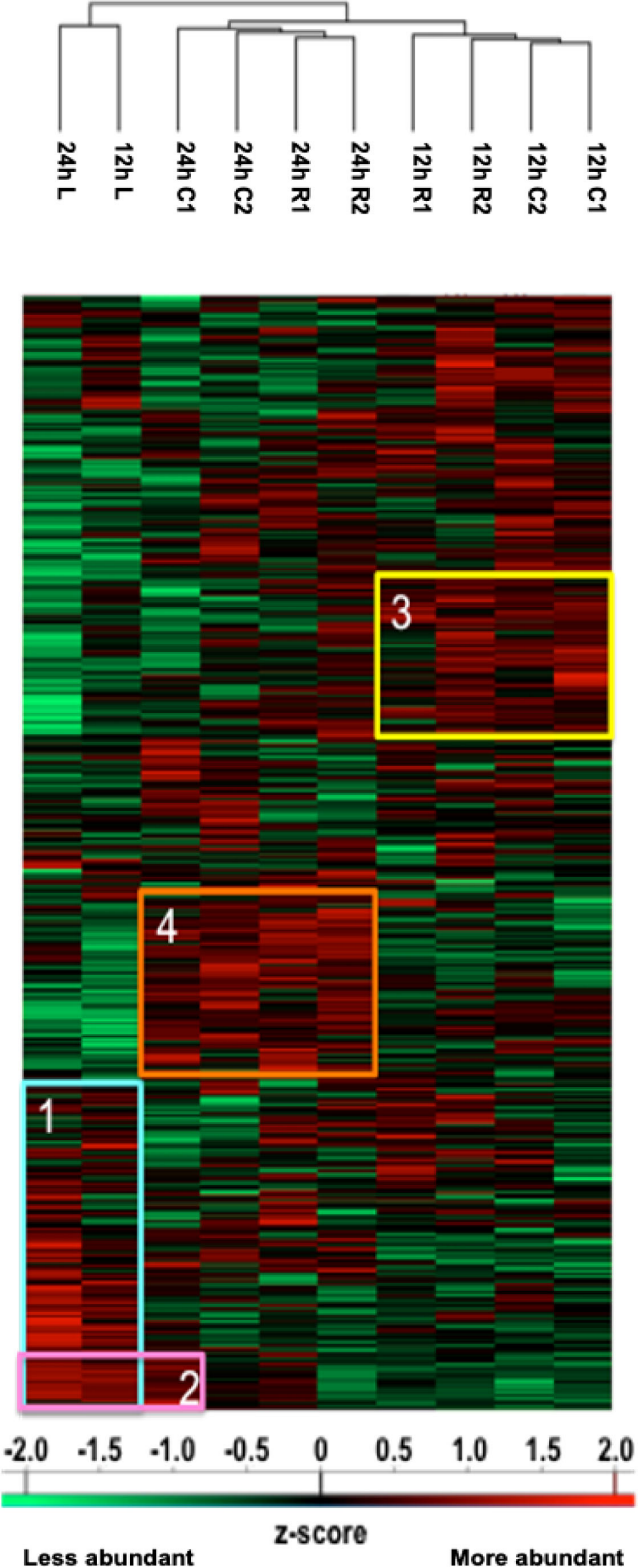
Heatmap illustrating hierarchical clustering obtained after analysis of digested peptides isolated in secretomes from spinal cord segments collected 12 h and 24 h after SCI. Four clusters are highlighted. Cluster 1 corresponds to the inflammatory proteins’ accumulation in lesions. Cluster 2 demonstrates that the proteomic profile of C1 starts to cluster with lesions after 24 h. Clusters 3 and 4, respectively, represent proteins more abundant in other segments at 12 h (neurite outgrowth inhibition) and at 24 h (synaptogenesis and neurogenesis proteins).

We studied the protein content of secretomes collected from Rostral 1 and 2 (R1, R2), lesion (L) and caudal 1 and 2 (C1, C2) spinal cord slices extracted 12 h or 24 h after SCI. After in solution digestion of secretome proteins, we proceeded to a nanoLC-HRMS in MS/MS mode analysis (**Supplementary Data 1**; **Figure 1** and **Table 1**). Collected data were analyzed, multi-samples tests were performed, and a hierarchical clustering was obtained (**Figure 1**). Two main branches detected. The first isolated lesion secretomes (12 h and 24 h) from secretomes of other segments. In lesion secretomes (Cluster 1), the more abundant proteins were inflammatory proteins (CRP, CXCL1, 2,3, interleukin 6), complement proteins (Cfi, C5, C1qc, C1r, C1s, C1qbp, C1q, C2, C3, C4, Cfp, C8g, C8a, C9, C6, Cs), calreticulin, metalloproteinase inhibitors 1 and 2, cathepsins B and D (**Figure 1** and **Table 1**). Of note, secretomes from C1 at 24 h clusterized with the secretomes of lesion segments, which confirmed and extended our previous data showing that, at later time points, lesion and C1 caudal segments harbored similar proteomic profiles (Cluster 2) (**Figure 1** and **Table 1**). Overexpression of complement components that were characteristics of innate immune response was initially restricted to the lesion segment before spreading essentially in the C1 segment between 12 h and 24 h and thus the acute phase of SCI-associated inflammation. Secretomes from other segments were partially clusterized depending on the time course and location. Thus, secretomes from rostral and caudal segments collected 12 h after SCI were enriched in neurogenesis inhibitors such as Nogo and neuroendocrine-specific protein (NSP) (Cluster 3) (**Figure 1** and **Table 1**). On the opposite, after 24 h, proteins promoting neurogenesis (Robo 1 and 2, Plexin B1, Semaphorins 6d and 4b, neuronal cell adhesion molecule 1 and 2 (Ncam1 and Ncam2)) were highly represented in secretomes from caudal and rostral segments (**Figure 1** and **Table 1**). It is especially interesting to notice that the intensity of the inflammatory response raised between 12 h and 24 h, as well as the proteomic profiles of rostral and caudal segments changed in this time window. Proteomic study performed during the acute phase at 12 h and 24 h also demonstrated the presence of immunoglobulins. Immunoglobulins found were IgG (IgG1, IgG2a, IgG2b, IgG2c) (**Supplementary Table 2**). Of note, IgGs were abundantly detected in spinal cord slices at early time points following SCI. We previously showed the presence of IgGs in the supernatants of SC slices at 3 days (3 D), 7 days (7 D), and 10 days (10 D) after SCI (4). Since B-cells were not detected in the spinal cord parenchyma during SCI we concluded that yet unknown mechanisms supported the uptake and subsequent release of IgGs from spinal cord slices (4). Their presence at such an early time (12 h) was surprising, and knowing that IgGs, depending on their subtype, have different receptors and functions, such as the ability to fix complement, we decided to focus our study on these immunoglobulins. We thus performed an in-depth analysis to further decipher the time-course of IgGs release from SC slices during SCI.

**Table 1:** Label free quantification (LFQ) values of proteins found in 12 h and 24 h secretomes.

Protein	Uniprot reference	12h R1	12h L	12h C1	24h R1	24h L	24h C1
**Immune Response**							
**C5**	**A0A096P6L9**	27.105	30.753	27.1991	29.8638	31.133	28.256
**Complement facteur I**	**A0A0G2K135**	28.6862	31.4865	29.1756	30.9452	31.9867	30.7474
**C1q C**	**P31722**	27.2059	27.5941	27.5216	28.0868	28.9265	27.5725
**C8g**	**D3ZWD6**	25.4009	29.3109	25.6515	28.2115	29.3498	27.933
**C6**	**F1M7F7**	26.2006	28.4164	25.2068	26.0326	28.8182	26.0147
**C9**	**Q62930**	29.3776	32.0729	28.8857	30.5028	32.8296	29.9725
**C9**	**Q62930**	29.3776	32.0729	28.8857	30.5028	32.8296	29.9725
**Cfb**	G3V615	27.5889	29.7907	28.1228	28.8318	30.2445	29.0034
**Cfd**	**P32038**	25.8039	29.2837	26.1685	27.3739	29.4617	26.6487
**C1s**	**G3V7L3**	25.9108	26.0894	0	0	26.1576	25.6268
**C1q B**	**P31721**	26.8409	27.8129	27.6312	27.3534	27.9999	27.1107
**C3**	**M0RBF1**	34.5677	35.9915	34.1207	35.4205	36.3782	34.9085
**C1q binding protein**	**O35796**	27.1542	28.7074	28.0595	28.183	28.7206	28.348
**C4**	**Q6MG90**	28.7578	31.2414	27.9086	29.5244	*21.1205*	30.2756
**Complement C1q A**	**P31720**	26.8299	28.2033	27.1038	28.0575	28.9744	28.2956
**C8**	**D3ZWD6**	25.4009	29.3109	25.6515	28.2115	29.3498	27.933
**C2**	**Q6MG73**	27.4799	29.0364	0	28.0699	29.841	27.8886
**C1q receptor**	**Q9ET61**	0	0	0	0	24.3473	24.4574
**CRP**	**P48199**	27.3319	29.1611	26.934	28.2882	29.9143	25.7745
**Cathepsin D (ctsd) protease**	**P24268**	29.5102	29.6845	30.016	29.7627	30.1024	29.5429
**Cathepsin B (ctsb) protease**	**P00787**	30.6649	31.0627	30.869	31.1476	31.5995	30.7585
**Metalloproteinase 1 inhibitor**	**P30120**	31.2912	31.3888	30.293	30.0952	32.0704	30.4234
**Metalloproteinase 2 inhibitor**	**P30121**	25.1333	26.2628	26.013	26.0498	26.7354	26.3374
**Axonal Guidance**							
**Roundabout homolog 1 Robo 1**	O55005	25.5019	25.6818	25.5182	25.4674	25.7368	26.0018
**Protein Robo2**	**Q9QZI3**	26.7878	25.2904	25.377	27.5075	26.8033	28.6504
**Neuronal cell adhesion molecule**	**A0A0G2K329**	32.237	32.0057	31.8815	32.527	31.8527	32.2182
**Neural cell adhesion molecule 1**	**P13596**	33.6315	33.5348	33.7206	34.7838	34.0443	35.0052
**Protein Ncam2**	A0A0G2K7P9	29.2571	28.2036	29.191	30.695	29.7085	30.0036
**Contactin 4**	Q62845	0	0	0	22.7805	0	24.6327
**Contactin 6**	P97528	0	0	0	24.2783	0	24.4514
**Semaphorin 6d**	A0A0G2JZC4	0	0	0	24.0672	0	24.8241
**Semaphorin 4b**	F1LSV0	26.4542	26.0691	26.504	26.7882	25.5816	26.8069
**Plexin B1**	D3ZDX5	28.3156	28.2094	28.5334	29.5902	27.9306	29.0632
		35_37	32_35	29_32	26-29	23-26	20-23	0

The color shading corresponds to the various LFQ values assigned after our protomics analysis.

**Table 2:** Sequence coverage in the SCI data from the different isotypes and regions of immunoglobulins from secretome 1 day after lesion.

Isotype	Coverage (%)
IgG1	51.23
IgG2A	66.46
IgG2B	41.74
IgG2C	34.65
IgM	31.52
Heavy V	53.96
Lambda C	93.27
Lambda V	39.34
Kappa C	41.51
Kappa V	79.51

### Meta-Analysis of the SCI Data

**Figure 2:**
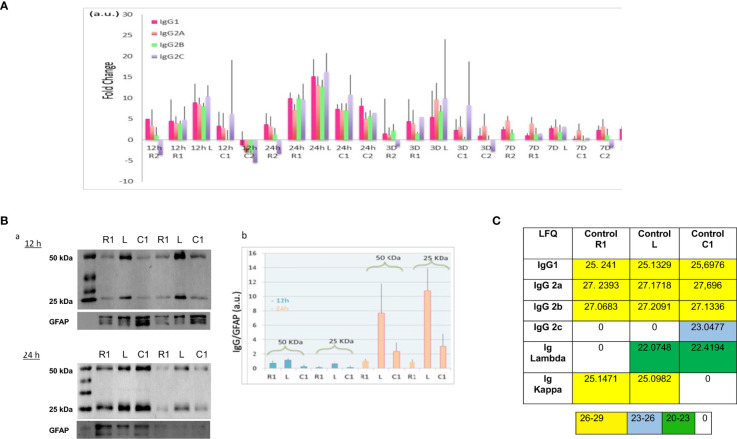
**(A)** Ratio of IgG according to time course after SCI and spinal cord segment. Rationalization was performed by comparison with the control (non-lesioned spinal cord). **(B)** (a) Western blot of 12 h and 24 h secretomes, with anti-immunoglobulin antibodies. (b) Histogram illustrating the immunoglobulin level in function of time and segment was obtained by ImageJ software. Results rationalization was carried out using GFAP. **(C)**. Inset **Table 3**. Immunoglobulin rates in control spinal cord. (LFQ: label free quantification).

Except in shark and camel, where minibodies have been identified as dimers of shortened heavy chain of immunoglobulins (13–16), most vertebrate species display antibodies composed of four polypeptidic chains, two heavy chains and two light chains, linked by disulfide bonds (17). Heavy chains are subdivided in four domains, Variable Heavy (VH) and Constant Heavy (CH1, CH2, and CH3), whereas light chains are subdivided in two domains, Variable (VL) and Constant (CL) (17). In the variable domains (VH and VL), hypervariable regions (CDR for complementary determining regions) and variable regions (FR for framework) are present, determining the paratope of the antibody. Meta-analysis performed on triplicate data from shot gun proteomic based on spatio-temporal study of spinal cord injury from 12 h to 10 days, gave evidence of IgGs isotypes with a sequence coverage of 48,52% (4, 5, 12). The most abundant were IgGs, especially IgG2a and IgG2B, but constant kappa light chain was also found with high LFQ values (**Supplemetary Table 2**). Indeed these immunoglobulins as well as the Lambda constant chain were detected in the secretome regardless of the time after lesion and the segment. Of note, some immunoglobulins (IgG1, IgG2a, IgG2b, constant kappa and lambda light chains) were also identified in the segments from the control group (**Supplemetary Table 2**). For IgG2a, the most part of the identified peptides whatever the conditions covered the CH2 and CH3, and one single peptide was detected in CH1 (**Supplementary Data 2**). For IgG2B, CH2 was mostly covered and one peptide in CH3 was found regardless of the time and the spatial localization (**Supplementary Data 2**). Concerning the Lambda chain, almost 93% of the constant part was identified (**Table 2** and **Supplementary Data 1** **).** It is interesting to note that IgM and IgG2c were also identified but almost exclusively at 12 h after the lesion. This means that these Igs were not natively present in the spinal cord and their release was therefore induced by the lesion (**Supplemetary Table 2** and **Supplementary Data 2**). Indeed, IgG2c and IgM-like proteins were mostly detected in secretomes from lesion and rostral segments at 12 h and then in lesion and caudal segments at 1 D. This fits with the slide from rostral to caudal segments previously demonstrated in whole proteomic studies (4). This time window is clearly unusual for IgM production which appears normally 6 to 7 days after trauma. We also observed that IgG2c diseapered afterward and IgM slightly decreased from 3 D and its production stayed centered at the lesion site. For IgM, different peptides covering various parts of the heavy chain were identified (**Supplementary Data 2**). At this time window (12 h, 24 h), we also identified a heavy variable part with a coverage of 54% (**Table 2** and **Supplementary Data 1**). At the lesion site 12 h after SCI, variable parts of Kappa and Lambda light chains were also detected. Lambda V then disappeared whereas Kappa V spread in all segments at 24 h and then disappeared. Only 40% of the VL were identified whereas almost 80% of the VK were characterized (**Table 2** and **Supplementary Data 2**). To assess more accurately the spatio-temporal evolution of immunoglobulin levels in the secretomes after SCI, their LFQ values were normalized to those quantified in the secretomes of controls (non-lesioned spinal cord) (**Figure 2A**). This confirmed that abundance of IgG1, IgG2a, IgG2b, and IgG2c increased between 5 to 15 times from 12 h to 24 h in Rostral 1, Lesion and Caudal 1 segments. Moreover, at 24 h, the levels of IgGs increased only in the Caudal 2 segment. These levels began to decrease from 3 days and became low at 7 days for all these IgGs. However, IgG2a had the tendency to remain higher than other IgGs from 24 h to 10 days. Moreover, this analysis revealed again that immunoglobulins showed a trend to be higher at the lesion site.

To complete our study, Western blot analyses with anti-IgG were carried out on secretomes of Lesion, Rostral 1, and Caudal 1 segments harvested 12 h and 24 h after SCI. These experiments were conducted in denaturing and reducing conditions (**Figures 2Ba**). Two biological duplicates were loaded on each gel. These experiments confirmed the presence of heavy and light chains detected at 50 kDa and 25 kDa, respectively. In gel digestion was also performed in parallel and validated the identity of these bands as heavy and light chains (**Supplementary Data 3**). Previously, during our proteomic analysis, we detected constant levels of GFAP in these secretomes (12). Therefore, we decided to use this protein as an internal control during our Western blot experiments. The intensity of heavy and light chain bands was quantified and normalized to those of GFAP. Even if no significant differences were found, heavy and light chains showed again a trend to be higher at the lesion site, especially at 24 h after SCI (**Figure 2Bb**).

### Immunoglobulins Are Detected in the Normal CNS

As described above, we identified immunoglobulins within the control spinal cords. This raised the question of the origins of these immunoglobulins released in the spinal cord under steady state conditions. The release of immunoglobulins from SC slices could be a consequence of the experimental setting since tissue slicing and subsequent short-term organotypic cultures are likely to alter the physiological behavior of neural cells. To address this issue, we extracted proteins from a freshly frozen intact spinal cord, thus avoiding any culture bias, and found similar results (**Figure 2C** and inset **Table 3**). Since neural cells express receptors binding the constant fraction (Fc) of immunoglobulins (18), one may propose that the presence of IgGs in spinal cord neural cells is primarily conditioned by their transport from blood to brain and their subsequent internalization and release by neural cells. However, intriguingly, a survey of the mouse brain atlas showed that mRNA expression of IgG1 and IgG2 classes in distinct CNS regions including the spinal cord, cerebellum, and hippocampus in both juvenile and adult mice (http://mouse.brainmap.org/) (**Supplementary Figure S1A**). Altogether these results support that immunoglobulins are present in the basal state in the spinal cord.

### Identification of Immunogobulins in SCI Tissues

**Figure 3:**
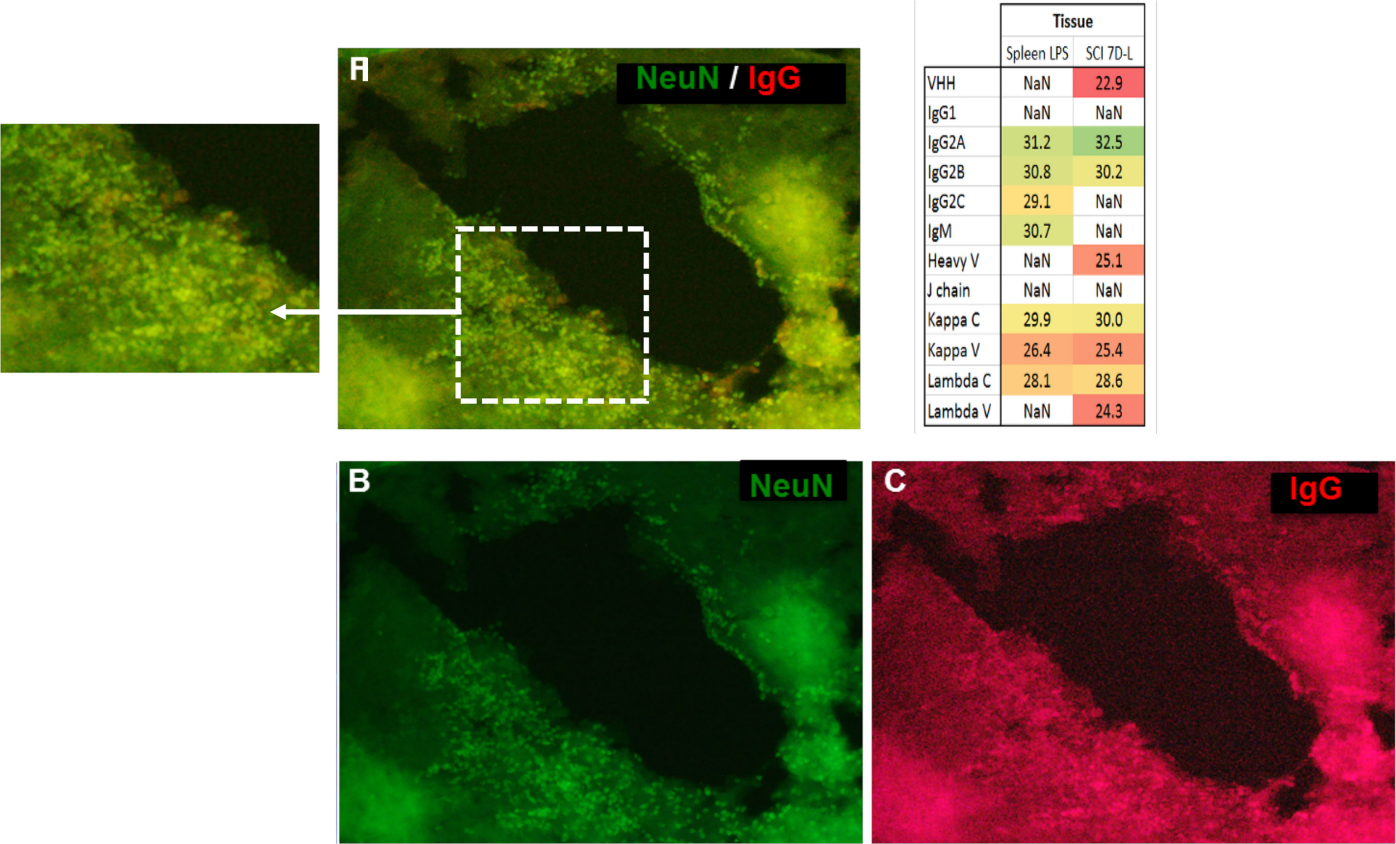
The immunofluorescence performed on a section of lesion segment from the spinal cord revealed a colocalization **(A)** between NeuN (**(B)**, green, Alexa Fluor® 488) and IgG (**C**, red, Alexa Fluor® 647) (20X objective). Inset **Table 4**. Sequence coverage in the SCI data from the different isotypes of immunoglobulins from SCI tissues at 7 days after lesion and spleen from rat stimulated with LPS as control.

Antibodies are mostly identified from biological fluids and mass spectrometry using shot gun proteomic (19) or top-down proteomic (20, 21) has highly contibuted in that direction. By contrast, immunoglobulins in complex samples such as tissues are more difficult to characterize. In order to further isolate and characterize IgGs in tissues, IgG enrichments may be necessary. Conventional enrichment of IgGs in complex samples is based on affinity chromatography with protein A, protein G, or antigen, and can be followed by mass spectrometry analysis (22). However, a new alternative technique named the Melon gel is also possible but it is mainly used to purify IgGs from serum. More precisely, the Melon gel is not an enrichment technique but is based on negative selection using a resin that captures all proteins except IgGs. To the best of our knowledge, this technique is used exclusively on serums and not on protein from tissue extracts (23–25). So it was decided to test Melon gel on protein extracts from tissue as well. Enrichment procedures using ammonium sulfate precipitation and Melon gel purification were performed on protein extracts from the spleen as a contol and a spinal cord lesion segment withdrawn seven days after injury (SCI 7D-L) (**Figure 3** and inset **Table 4** and **Supplementary Data 3**). After the MS analysis with MaxQuant, IgG were detected, confirming the efficiency of the enrichment procedure. Constant Lambda and Kappa light chains, as well as Kappa V were detected in both the spleen and SCI 7D-L as we previously found in secretome. Concerning immunoglobulin heavy chains, only IgG2A and IgG2B were detected in SCI 7D-L (**Figure 3**; inset **Table 4**). To extend our study, we validated the presence of immunoglobilins in SCI tissue by immunofluorescence (**Figure 3**). Co-labeling was performed with anti-NeuN (neuronal marker) and anti-IgG on sections of lesion segments from spinal cord collected 24 h after lesion (**Figures 3A–C**). This revealed that cells around the lesion site stained with anti-IgG were neurons since they were also labelled with anti-NeuN. We previously demonstrated the impact of RhoA pharmacological inhibition on neurites outgrowth and synaptogenesis (12). This was confirmed by shotgun proteomics performed on rostral, caudal, and lesion segments collected 12 h after lesion with or without treatment with RhoA inhibitor (**Supplementary Figure 3**). This revealed that inflammatory proteins and immunoglobulins were more abundantly present in the lesion site with or without RhoAi treatment (Cluster 1). By contrast, Cluster 2 showed proteins involved in synaptogenesis which were more highly detected after RhoAi treatment. Cluster 3 represented proteins involved in neurite outgrowth such as CNTF, contactin, neural cell adhesion molecule L1, Stathmin, Neurofascin, Neurotrimin, and Dynactin. These proteins were more abundantly detected in R1 and C1 segments without RhoAi treatment. We therefore evaluated at an early stage after SCI, the impact of such RhoA inhibition (RhoAi) on IgG levels released from spinal cord explants (**Supplementary Data 5**).

### RhoA Inhibition Increased IgG Release From Spinal Cord Explants

**Figure 4:**
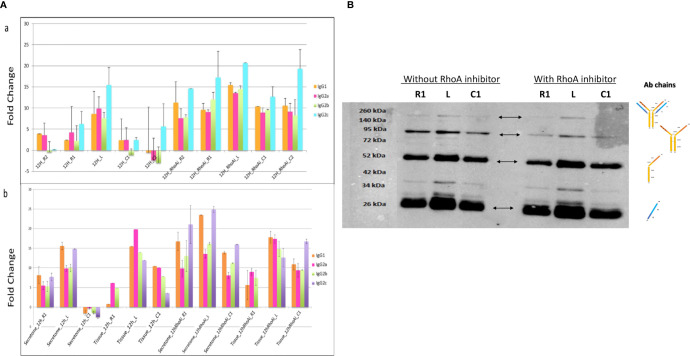
**(A)** (a) Fold change between IgG isotypes in secretome from the different segments collected 12 h after SCI and treatment with RhoA inhibitor. (b) Fold change of IgG isotypes between tissue and secretome. **(B)** Detection by Western blot of IgG chains in secretomes from rostral, caudal, and lesion segments collected 12 h after lesion and treatment with RhoA inhibitor.

RhoA inhibitor was injected at the lesion site at the time of lesion and SCI explants were collected at an early stage after SCI, that is, 12 h. A shot gun proteomic analysis was then conducted on secretomes from the various segments (**Figure 4Aa**). The LFQ values obtained for IgG were compared to those measured in secretomes of SCI explants from rats untreated with RhoA inhibitor. This revealed that RhoAi treatment increased the release of the various IgG isotypes considered in this study. In section R2, C1, and C2, fold changes reached 3 to 4, whereas in R1 and lesion fragment, fold changes reached 2 to 3. Western blot analysis in reducing conditions reconfirmed the presence of IgG in these secretomes (**Figure 4B**). Afterward, semi-quantitative analysis of IgGs in spinal cord tissue sections confirmed the impact of RhoAi treatment in R1, C1, and lesion segments (**Figure 4Ab**). Interestingly, while IgG1 and IgG2C were the most prevalent isotypes in the spinal cord secretomes of untreated injured animals, IgG2A was the most abundant IgG isotype in the spinal cord secretome of RhoAi-treated injured animals, especially at the lesion site (**Figure 4A**). Besides IgG2A, we also observed an isotype-specific effect of RhoAi treatment for other IgGs. After treatment with RhoA inhibitor, IgG2C as well as IgG1 highly increased in the secretome of the lesion segment while conversely IgG2C was not detected in R1, decreased in the lesion and was predominant in C1. IgG2A greatly increased in the lesion (**Figure 4A**). These results indicate that RhoAi treatment induced an isotypic commutations (systemic or local) and/or an isotype-specific mechanism of capture and release of IgGs by neuronal cells.

### Neurons Produce IgG

**Figure 5:**
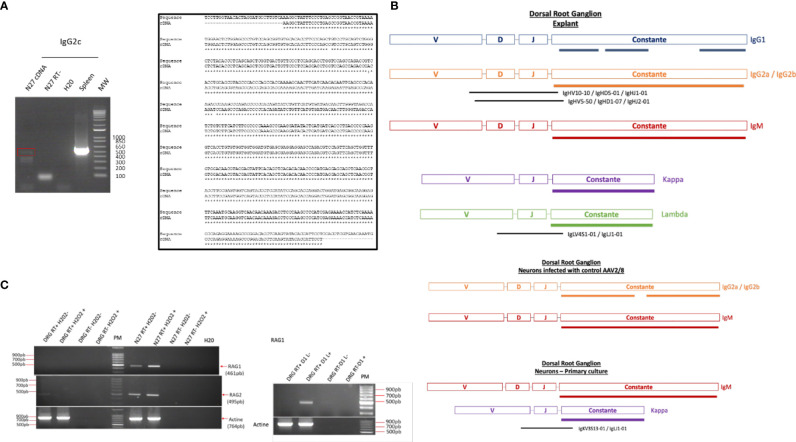
**(A)** RT-PCR amplification of IgG2c in spleen and N27 cells was confirmed by sequencing. **(B)** Reads coding immunoglobulin constant heavy and light chains as well as variable parts were identified in DRG explant or DRG primary neurons by survey on RNaseq data B (**Supplementary Data 4** and **Supplementary Table S1**) **(C)** Amplification of *Rag1* and *Rag2* mRNA in DRG and N27 cells was carried out by RT-PCR in presence of H_2_O_2_ to induce neuronal death, as observed during the spinal cord injury. Since expression of RAG1 encoding gene was not observed under these conditions, DRG cells were cultivated in the presence of secretome of lesion segment from 1 day after SCI following by RT-PCR. As a control to exclude genomic DNA contamination, the experiment was also carried out on a negative reverse transcriptase sample (RT-), H_2_0, negative control.

To determine if these IgG were synthesized or recaptured by neurons, a transcriptomic approach was undertaken in dorsal root ganglia (DRG) ND7/23 cell line stimulated with LPS to mimic an inflammatory condition as observed at the lesion site. Moreover, the same approach was performed in the non-differentiated N27 dopaminergic neural cell line (N27). Transcripts from the spleen serve as a positive control. By RT-PCR, we amplified the whole IgG2c ORF in N27 cells as another source of neural cells. Cloning was performed and sequencing confirmed that this gene was expressed in N27 cells demonstrating the neural origin of these immunoglobulins (**Figure 5A**). These results are in line with recent data showing the expression of the genes encoding IgG3 and IgM in murine spinal and supraspinal neurons (26). Allen Brain Atlas confirms in mouse the presence of IgG3 and IgH-VS107 immunoglobulin chains in the cortex and hippocampus regions (**Supplementary Figures 1A, B** **)** or spinal cord (**Supplementary Figure 2**). Analyses of RNAseq Sequence Read Archive data from rat DRG neurons confirmed the expression of immunoglobulins heavy and light chains (constant and variable) (**Figure 5B**; **Supplementary Table 1** and **Supplementary Data 4**). We identified constant IgG1, IgG2(a, b), and IgM heavy chains as well as Kappa and Lambda light chains in DRG explant. IgG2(a, b), IgM, and Kappa chains were also detected in primary culture of DRG neurons. Several V(D)J chains were also identified associated with constant IgG2(a, b) chains and Kappa chains (**Figure 5B**). Taken together, the presence of immunoglobulins in neurons is comforted. Moreover, it is well known that the proteins of the immunoglobulin superfamily (IgSF) are implicated in various stages of brain development, including neuronal migration, axon pathfinding, target recognition, and synapse formation as well as the maintenance and function of neuronal networks in the adult (27). To support Ig production by neurons, we studied in DRG and N27 cells the expression of genes coding the RAG1 and RAG2 enzymes controlling V(D)J recombination. Since during SCI or other brain trauma, an important amount of neuronal death is observed, we thus mimic this process by using H_2_O_2_ treatment (28). Under that condition, *Rag1* expression was only observed in N27 cells whereas *Rag2* mRNA was detected in both cell lines (**Figure 5C**). Since *Rag1* expression may be induced by specific factors produced after SCI, DRG cells were cultivated in the presence of secretome of the lesion segment collected 1 day after lesion representing the acute phase of inflammation after SCI (**Figure 5C**) (12). In such conditions, *Rag1* expression was observed. These results suggest that neurons produce a variability of immunoglobulins through V(D)J recombination.

### Identification of FcγRs (CD16, CD32) in DRG ND7/23 Neurons

**Figure 6:**
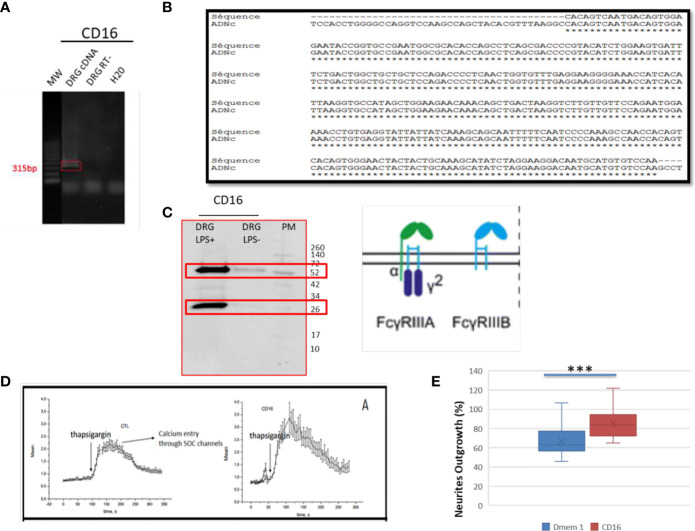
Identification of FcγRIII receptors in sensory neurons. **(A)** RT-PCR amplification of *Cd16* mRNA. **(B)** Sequence alignment of the nucleic acid sequence. **(C)** Western blot analysis to detect CD16 in DRG cells treated with LPS. **(D)** CD16 activation by specific antibody in TG-activated SOCE conditions impacts Ca^2+^ homeostasis. Quantification of the results are presented in the left panel for the controls and in the right panel for CD16 activation. **(E)** Neurite outgrowth in DRG ND7/23 cells in the presence of anti-CD16 activating CD16. ***P value of < 0.001.

**Figure 7:**
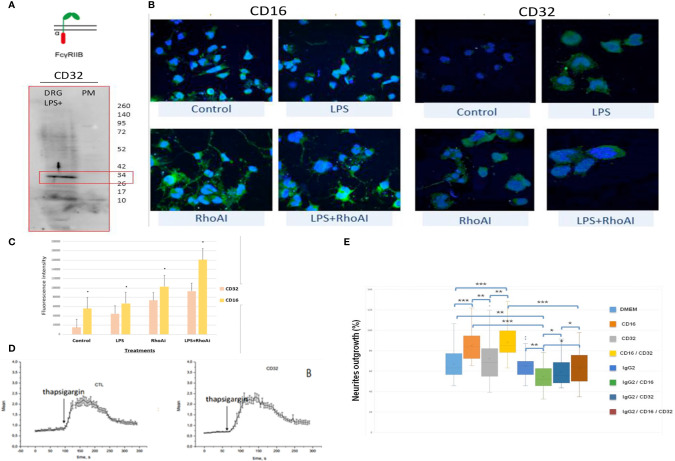
FcγRIIB receptor identification in sensory neurons. **(A)** Western blot revealing CD32b receptor in DRG cells. **(B)** Immunofluorescence performed with anti-CD16 or anti-CD32 on DRG cells stimulated with LPS and/or RhoAi. Nuclei were counterstained with DAPI (blue). **(C)** Quantification of CD16 and CD32b immunofluorescence in DRG ND7/23 cells after RhoAi and/or LPS treatments. **(D)** CD32b activation by specific antibody in TG-activated SOCE conditions does not impact Ca^2+^ homeostasis. Quantification of the results are presented in the left panel for the controls and in the right panel for CD32 activation. **(E)** Neurites outgrowth in DRG ND7/23 cells in the presence of specific CD16 or CD32b activating antibody or CD16 and CD32b activating antibody with or without anti-GFAP. Mouse anti-GFAP displayed an IgG2 isotype and served as a control of Fc gamma receptors activation. *P value of < 0.05, **P value of < 0.01, ***P value of < 0.001.

In the immune system, immune response modulation by IgGs involves the antibody-dependent cell-mediated cytotoxicity (ADCC) mechanism (29). In this context, IgGs through their paratopes recognize their target and label the cells to be destroyed. For this purpose, cytotoxic immune cells recognize the bound IgGs through their constant region. This recognition involves FcγR such as CD64, CD32, and CD16 expressed at the cell surface of cytotoxic cells. For a long time, it was considered that the expression of these receptors was restricted to immune cells. However, recent studies have demonstrated the presence of FcγR I (CD64) at the cell surface of glial cells such as microglia and astrocytes (30). More recently, Zhang and collaborators also established that FcγRI, but not FcγRII (CD32) and FcγRIII (CD16), was also expressed in a subpopulation of primary sensory neurons (31). Allen Brain Atlas indicated that Igs are colocalized with CD16 and CD32b in the same mouse brain regions (**Supplementary Figures 1C, D**). At a first step, to demonstrate the expression of CD16 and CD32 receptor, we used transcriptomic and Western blot approaches in DRG ND7/23 cells (**Figure 6**). A RT-PCR experiment was carried out to amplify *Cd16* transcripts. A positive fragment was obtained, and sequencing confirmed the expression of this gene in DRG ND7/23 cells (**Figures 5A**, **6B**). These results are in line with RNAseq analyses performed on rat DRG (**Figure 5** and **Supplementary Table 1**). To confirm the expression of CD16 at the protein level, Western blot experiment was conducted after immunoprecipitation with an anti-CD16 performed on protein extracts from DRG ND7/23 cells stimulated or not with LPS (**Figure 6C**). LPS stimulation was used to mimic an inflammatory condition as observed during SCI. In both conditions, a band close to 30 KDa and corresponding to CD16 was revealed. An additional band close to 52 KDa was also observed. It has been described that efficient cell expression of CD16 requires its interaction with a dimer of Fcer1g (29). Fcer1g mass is close to 10 KDa. Therefore, the band around 52 KDa observed in our experiment may correspond to CD16 bound to Fcer1g dimer. Altogether, this confirmed the expression of CD16 in DRG ND7/23 cells. Interestingly, under LPS treatment the intensity of the bands increased compared to the control condition. This pinpointed that the inflammatory environment mimicked by LPS regulated CD16 expression in sensory neurons (**Figures 6C**). To test the activation of CD16 with anti-CD16, intracellular calcium release (32) was registered (**Figure 6D**). The result obtained confirmed its activation. To identify the CD32b receptor in DRG cells, Western blot analysis was also conducted and revealed a band at 34 KDa matching with its molecular weight (**Figure 7A**). This receptor is known as the only inhibitory receptor of the FcγR family since it’s the only one to display the ITIM motif on its cytoplasmic domain. These results are in line with RNAseq data (**Supplementary Table 1**). Moreover, we observed that RhoA inhibition increased IgG release by SCI explants (**Figure 7A**). In macrophages, it has been shown that CD32b expression was also increased by LPS treatment (33). Therefore, to determine if RhoA inhibition treatment combined with LPS could modulate the expression of CD16 and CD32 in DRG ND7/23 cells, we studied their expression profile by immunofluorescence (**Figure 7B**). Under LPS and/or RhoAi treatments, the intensity of CD16 immunostaining increased remarkably compared to the control condition. In fact, this intensity was 4%, 40%, and 63% higher for LPS, RhoAi, and the combined LPS+ RhoAi treatments, respectively. In comparison to the control condition, the intensity of CD32 staining was also enhanced by 52%, 72%, and 88% for LPS, RhoAi, and LPS+ RhoAi treatments, respectively (**Figure 7B**). These results suggested the potential involvement of those factors in the upregulation of CD16 and CD32 expression. Moreover, even if anti-CD16 and anti-CD32 were employed at the same concentration, the detection threshold for anti-CD32 was lower. Indeed, in comparison with anti-CD32, the intensity of fluorescence measured with anti-CD16 was 67%, 33%, 30%, and 38% more intense for the control, LPS, RhoAi, and LPS+ RhoAi treatments, respectively (**Figure 7B**). This showed that the expression of CD16 was higher than CD32 under both basal and stimulated conditions. Since RhoAi modulated neurite outgrowth, synaptogenesis (12), and increased IgG release in the spinal cord as well as the expression of CD16 and CD32 in the sensory neurons (**Figures 7B, C** **)**, we investigated the possibility that activation of these Fc receptors in DRG ND7/23 cells could modulate neurite outgrowth.

### Antibody Dependent Neurite Outgrowth Modulation (ADNM) Responses

**Figure 8:**
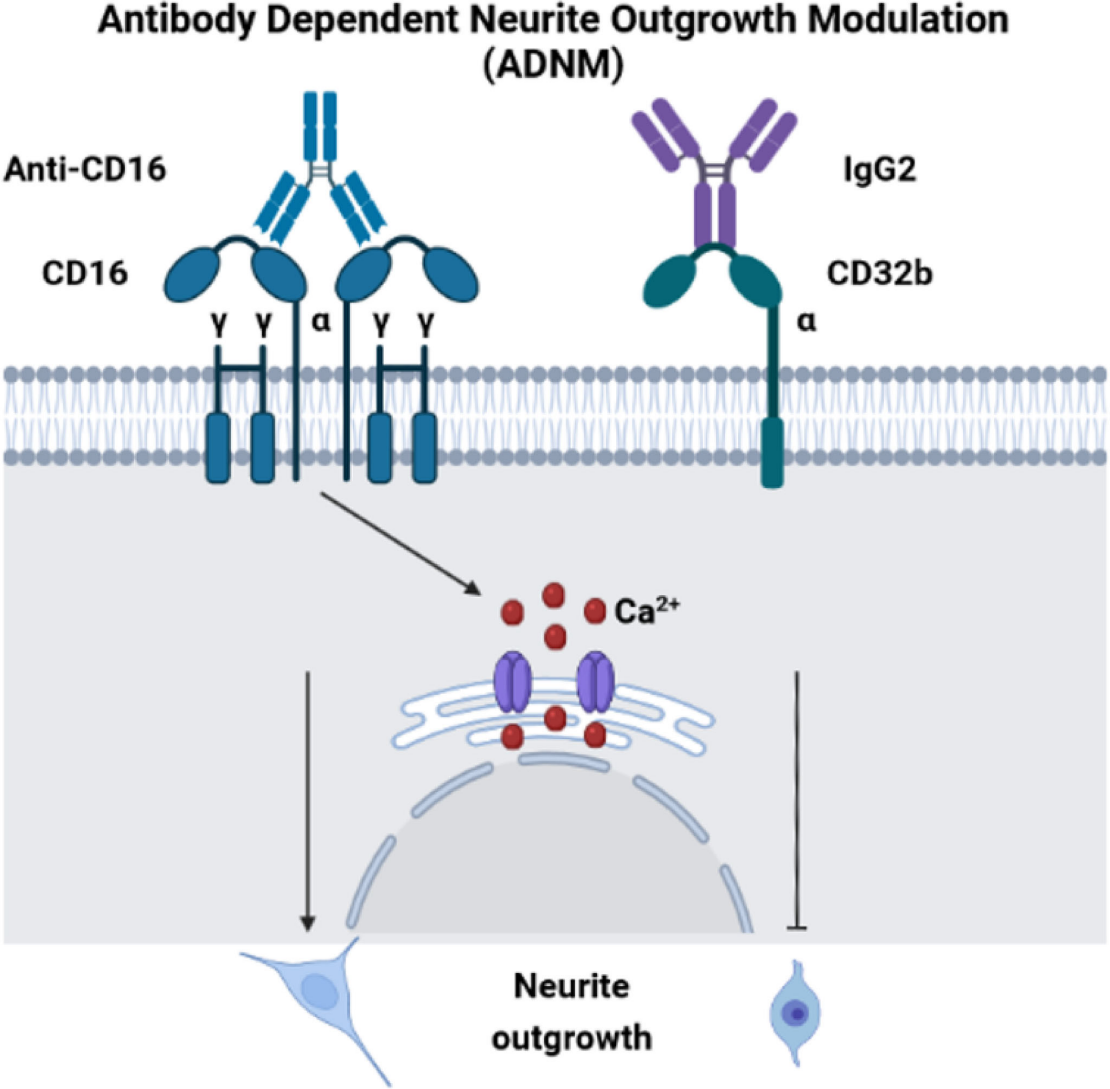
Antibody dependent neurite outgrowth modulation (ADNM). CD16 activation with anti-CD16 induces calcium release from RE and triggers neurite outgrowth. On the contrary, C32b activation by IgG2 isotype prevents CD16 from triggering neurite outgrowth.

Before performing neurite outgrowth assays, we confirmed that unlike anti-CD16 (**Figure 6D**), anti-C32 did not stimulate intracellular calcium release (**Figure 7D**). To test the impact of CD16 and CD32b activation with their specific antibodies on neurite outgrowth, DRG sensory neurons were incubated with anti-CD16 and/or anti-CD32 in the presence of IgG2 isotype (**Figure 7E**). In comparison with the DMEM control condition, the percentage of neurite outgrowth was higher after CD16 activation while CD32b activation had no effect. Moreover, activation of both CD16 and CD32b also led to an increase of the percentage of neurite outgrowth. Keeping in mind that CD32b is known as the only inhibitory receptor of the FcγR family, this showed that CD16 activation could prevail and be sufficient to overcome a potential inhibitory effect exerted by CD32b. To test if CD32b could exert such an inhibitory effect on neurite outgrowth, a stronger activation of CD32b was triggered by the addition of a treatment with IgG2 isotype (**Figure 7E**). In its presence, we observed that whatever the conditions, the percentage of neurite outgrowth remained close to the one observed in control conditions with DMEM or IgG2 treatment. In comparison with CD16 activation combined with CD32b activation, the percentage of neurite outgrowth was significantly reduced when DRG cells were incubated in the presence of IgG2 isotype + anti-CD16 +/- anti-CD32. Therefore, the activation of CD32b by IgG2 isotype prevented CD16 from triggering neurite outgrowth. Altogether, these results suggest that various IgG according to their isotypes could modulate neurite outgrowth through the activation of CD16 and CD32b. We named this process antibody dependent neurite outgrowth modulation (ADNM) (**Figure 8**).

## Discussion

Autoimmune diseases affect about 5%–7% of the world population and 3% of these autoimmune diseases involve pathogenic CNS-reactive autoantibodies (34). Neural repertoires, defined as the whole of neural populations and synaptic circuits supporting the cognitive and sensorimotor functional repertoires (35), express brain autoantigens such as myelin and synapse-derived proteins (glutamate decarboxylase (GAD2), acetylcholine receptor (AchR), and acetylcholine esterase (AchE)) (36). Interestingly, some brain autoantigens involved in major CNS functions are targeted in non-CNS autoimmune disorders. Such autoantigens, proposed to be named brain super autoantigens (35), notably include HSP60 and TROVE2, which are two major autoantigens in rheumatoid arthritis and lupus, respectively. HSP60 (*hspd1*) plays a major role in the control of synaptic neurotransmitter release and mutations in *hspd1* gene are responsible for autosomal recessive spastic paraplegia disease (37). Similarly, SNP variants of *trove2* gene (also knowns as RNA binding protein Ro60 autoantigen) have been associated with higher emotional memory capacity in healthy human subjects (36). It is thus thinkable that pathological autoimmunity in general is preferentially targeted toward brain autoantigens and reflects the distortion and/or the amplification of a physiological process in which autoimmunity supports cognition and CNS homeostasis (35). Favoring this view, in multiple sclerosis (MS) disease, a recent clinical study on 176 patients identified a large panel of autoantibodies recognizing more than 30 neuronal or glial autoantigens (38). Similarly, a number of neuropsychiatric disorders like autism spectrum disorder are accompanied by the synthesis of CNS-reactive antibodies (39). Independently from any consideration on their actual role, autoantibodies are thus emerging as a new generation of biomarkers in CNS disorders, ranging from neurotrauma to neuropsychiatric or neurodegenerative diseases (39). Interestingly, although brain-derived autoantigens might not be immunogenic under physiological conditions, several pathology-associated events might drive CNS-targeted autoimmunity. These notably comprise the occurrence of immunogenic post-translational modifications and the co-release by neural cells of brain autoantigens and danger signals. In TBI, autoantibodies are directed against a repertoire of CNS self-antigens (proteins) including GM1 gangliosides, myelin-associated glycoprotein, α-amino-3-hydroxy-5-methyl-4-isoxazolepropionic acid (AMPA), and N-methyl-D-aspartate (NMDA) glutamate receptors, and β-III-tubulin and nuclear antigens (34, 39–41). Moreover, immunocytochemistry studies performed with monoclonal antibodies as well as fragment antigen binding (Fabs) at the level of the CNS have shown the presence of immunoglobulin isotypes (IgG1, 2A, 2B, 2C), Kappa and Lambda chains (42, 43). In normal rat brain, IgG are detected, and this level reached a higher amount after head injury (44). *In situ* hybridization experiments depicted in mouse Brain Atlas also revealed the presence of such immunoglobulins encoding transcripts (IgG and IgM) in hippocampus, cortex, and spinal cord. Moreover, during spinal cord injury (SCI), traumatic brain injury (TBI) and a multitude of other CNS pathological conditions such as multiple sclerosis (MS), an intrathecal synthesis of IgGs is observed (45). The origin of such intra-CSF (cerebrospinal fluid) IgGs is still debated. The two main current hypotheses are that IgGs are produced locally by CNS-invading B-cells or are somehow transported from blood to CNS. In several neuroinflammatory pathological disorders, including notably MS, a hallmark of intrathecal IgGs is to form oligoclonal bands (OCBs), which suggests a rather limited diversity of antibodies and, consequently, a finite number of corresponding targeted antigens. While MS pathology is essentially characterized by the existence of multifocal inflammatory and demyelinating lesions (the MS plaques), OCBs develop in the quasi-absence of B-cells in inflammatory lesions. Indeed, OCBs are considered the products of clonally expanded B cells in the cerebrospinal fluid (CSF), representing the sum of contributions from B cells in the brain. However, large amounts of IgGs can be eluted from MS plaques in which lymphocytes are absent. Interestingly, in a recent study analyzing CSF samples from 115 MS patients, it was calculated that 3.2 billion lymphocytes would be necessary to generate such large amounts of intrathecal IgG levels (30 mg in 500 ml CSF) (46). CSF-circulating lymphocytes were estimated to account for <0.1% of the IgG levels in MS brains. Such a finding is thus compatible with the possibility that intrathecal IgGs might not derive from intrathecal B-cells (46). Supporting this fact, the neuronal distributions of IgG are found in motor neurons in brainstem nuclei including trigeminal motor nucleus, facial nucleus, and hypoglossal nucleus (43) and confirmed recently by transcriptomics analyses in mice spinal neurons (26) as well as our immunoflurorescence experiments (4) on rat spinal cord. The present study is in line with these results. Our proteomic data establish that IgG2A and IgG2B are present in the spinal cord in control and the level is similar in the time course of SCI. On the contrary, the protein related to IgM is only expressed 12 h to 24 h after lesion. This time window is not compatible with a B cell production since IgM are produced 7 to 10 days after antigen recognition. Accordingly, we demonstrated the expression of IgG2 isotypes encoding genes through RT-PCR experiments performed on CDNA from rat N27 dopaminergic neural cell line and data reuse of RNAseq datasets obtained from rat primary culture of DRG neurons. While a pathogenic role has been generally assigned to CNS autoantibodies, a neuroprotective function of natural IgG autoantibodies has also been suggested. In this line, several studies have shown that binding of such antibodies to the surface of neurons through FCγR enhances remyelination and modulate neuronal apoptosis (47–52). Consistently, our results demonstrate that CD16 (FcγRIII) activation also modulates neurite outgrowth. Taken together, all these data show that autoantibodies have two sources of production, peripheric, that is, B-cells and central. However, our results suggest that these autoantibodies may not only be involved in immune responses like those of B-cells but may also modulate neurite outgrowth. In this context, the crosstalk play between CD16 and CD32b activation in neurite outgrowth process evoked an antibody dependent neurite outgrowth modulation (ADMN). This mechanism could be seen as a counterpart of the antibody dependent cell cytotoxicity (ADCC) observed during the inflammatory response and involving the same receptors. Indeed, while CD16 activation favors neurite outgrowth, CD32b activation inhibited it. The balance between activation and inhibition could be fine-tuned by the nature of the various IgG isotypes released after trauma as we observed during SCI. Moreover, we cannot also exclude that among the immunoglobulin released, some could be involved in inflammation since the VHH found at 12 h presents sequence homology with a nanobody directed against RON protein. RON is a macrophage-stimulating protein receptor and is related to c-MET receptor tyrosine kinase. In the brain RON is expressed on tissue-resident macrophages including microglia (53). An *in vivo* deletion of the ligand binding domain of Ron (Ron−/−) promotes inflammatory (M1) and limits a reparative (M2) macrophage activation. These results are in line with its expression found at 12 h at the lesion site (54). The possiblitiy that anti-RON could activate microglia 12 h after lesion also fits with previous data obtained on microglia activation studies (5). These results can also be correlated with previous studies performed by the Popovich group, which showed that mice lacking heavy chain of immunoglobulins (IgH-6 KO) have an improved locomotor function and reduced spinal pathology compared to wild-type mice after SCI, suggesting a pathogenic role for antibodies (40, 55). The intraspinal pathology caused by B cells in wild-type mice is due in part to antibody-mediated ligation of Fc receptors and complement activation. But even though intraspinal B cell clusters and autoantibodies are maintained indefinitely in injured mouse spinal cord, there is no proof that these immune responses cause protracted neurological deterioration. A precipitous decline in function would be registered in both mice as B cells became activated and autoantibodies were synthesized. B cells and autoantibodies would respond to proteins that are newly expressed in growing axons, remyelinating oligodendrocytes, stem cells, or new endothelia. In that case, little or no additional gain of function beyond that achieved prior to the onset of the autoimmune response would be expected, which is not the case. In fact, in animal models of SCI, spontaneous recovery of function is registered after a period of weeks or months post-injury. Taken together, results in wild-type animals tend to show that autoantibodies are not only involved in pro-inflammatory response but also in neuroregeneration. Unfortunately, the IgH-6 KO model fails to confirm this hypothesis because the invalidation blocks the two possible sources of IgG production in the same manner if the IgGs are synthesized by the same mechanism (VDJ recombination). All this evidence supports that IgGs and their receptors are involved in neurite outgrowth modulation. This interesting change of paradigm constitutes a ground-breaking advance in the field of both neuroscience and immunology. The story appears even more complex since CNS immunoglobulins seem to have various cellular origins. Indeed, the diversity of immunoglobulins identified during our study, that is, reuse of RNAseq datasets obtained from DRG explants and proteomics analysis performed on injured spinal cord, does not fit with the restricted number of IgG isotypes found in DRG sensory neurons. In line with such an assumption, our previous immunofluorescence experiment performed on injured spinal cord slices also revealed a staining in astrocytes (4, 12). A survey of “Geodatasets” (https://www.ncbi.nlm.nih.gov/gds/), the NIH-compiled bank of mRNA expression studies, focused specifically on the mRNA profile of spinal cord astrocytes under inflammatory conditions confirmed that astrocytes were a second source of neural IgGs. Indeed, we found one relevant study in which astrocytes derived from mice suffering from experimental allergic encephalomyelitis were cell-sorted at different time points and assessed about their RNA profiles. Surprisingly, when we reused the supplementary data provided in this work by Itoh and collaborators we found that the mRNA species showing the highest fold changes in EAE spinal astrocytes as compared to control spinal cord astrocytes were indeed mRNAs coding for IgG2c and kappa chains or junction chains (56) (**Supplementary Figure 4**). Now the challenge is to clarify the function of these CNS immunoglobulins.

## Data Availability Statement

The datasets used for analysis and the annotated MS/MS spectra were deposited at the ProteomeXchange Consortium (http://proteomecentral.proteomexchange.org) via the PRIDE partner repository with the dataset identifier PXD004639.

## Ethics Statement

All studies on animals were reviewed and approved by the institutional committee for the protection of animals of the Slovak University of Science and conformed to the European Directive 2010/63 on the use of the animals for research purposes, as well as with the Slovak animal welfare laws Nos. 377/2012 and 436/2012.

## Author Contributions

Conceptualization, MS. Methodology, AC, M-AK, CM, SO, FA, FR, DC, IF, and MS; Software MS; Validation, M-AK, DC, and MS; Formal analysis, MS and DC. Investigation, DC and MS; Resources, DC, IF, and MS; Data curation, MS, AC, M-AK, and SO; Writing – Original Draft MS. Writing - Review and Editing, IF, FR, DD, DC, and MS; Supervision, FR, DC, and MS; Project Administration, DC and MS; Funding Acquisition, DC, IF, and MS. All authors contributed to the article and approved the submitted version.

## Funding

This research was supported by funding from Ministère de l'Enseignement Supérieur, de la Recherche et de l'Innovation (MESRI), Institut National de la Santé et de la Recherche Médicale (Inserm), I-SITE ULNE (Nobody Project), and Université de Lille. The authors would like to thank Raphaël Decaudin for his contribution to this manuscript.

## Conflict of Interest

The authors declare that the research was conducted in the absence of any commercial or financial relationships that could be construed as a potential conflict of interest.

## Publisher’s Note

All claims expressed in this article are solely those of the authors and do not necessarily represent those of their affiliated organizations, or those of the publisher, the editors and the reviewers. Any product that may be evaluated in this article, or claim that may be made by its manufacturer, is not guaranteed or endorsed by the publisher.

## Supplementary Material

Supplementary Table 1 Sequence read archive data from rat dorsal root ganglion explant and primary culture of DRG neurons revealed the expression of genes coding immunoglobulin heavy and light chains as well as cluster of differentiation markers.

Supplementary Figure 1
**(A)** *IgG3*, **(B)** *IgH-Vs107*, **(C)** *Cd32b*, and **(D)** *CdD16* mRNA expression in the hippocampus and in the cerebellum of mice according to the Allen Brain Atlas, mRNA expression.

Supplementary Figure 2
**(A)** *IgG3* mRNA expression in the spinal cord; **(B)** of mice according to the Allen Brain Atlas, mRNA expression.

Supplementary Figure 3 Heatmap illustrating hierarchical clustering obtained after analysis of digested peptides isolated from rostral, caudal, and lesion segments collected 12 h after lesion and treatment with RhoA inhibitor. Cluster 1 corresponds to the inflammatory proteins and immunoglobulins. Cluster 2 contains proteins involved in synaptogenesis, and Cluster 3 proteins are involved in neurite outgrowth.

Supplementary Figure 4 Rodent mRNA species after NGS sequencing showing the highest fold changes in EAE spinal astrocytes as compared to control spinal cord astrocytes *IgHa*, *Ig Kappa c*, *J Chain*, and *Cd32b* mRNA have been identified in astrocytes from the spinal cord and the cortex.
