# Supplementary material for: The Antibody Dependant Neurite Outgrowth Modulation Response Involvement in Spinal Cord Injury
Source: Front Immunol. 2022 Jun 16;13:882830. doi: 10.3389/fimmu.2022.882830 (PMC9245426; doi:10.3389/fimmu.2022.882830)
Supplement: Supplementary Figure 1 — (A) IgG3, (B) IgH-Vs107, (C) Cd32b, and (D) CdD16 mRNA expression in the hippocampus and in the cerebellum of mice according to the Allen Brain Atlas, mRNA expression. [file Presentation_1.pdf]

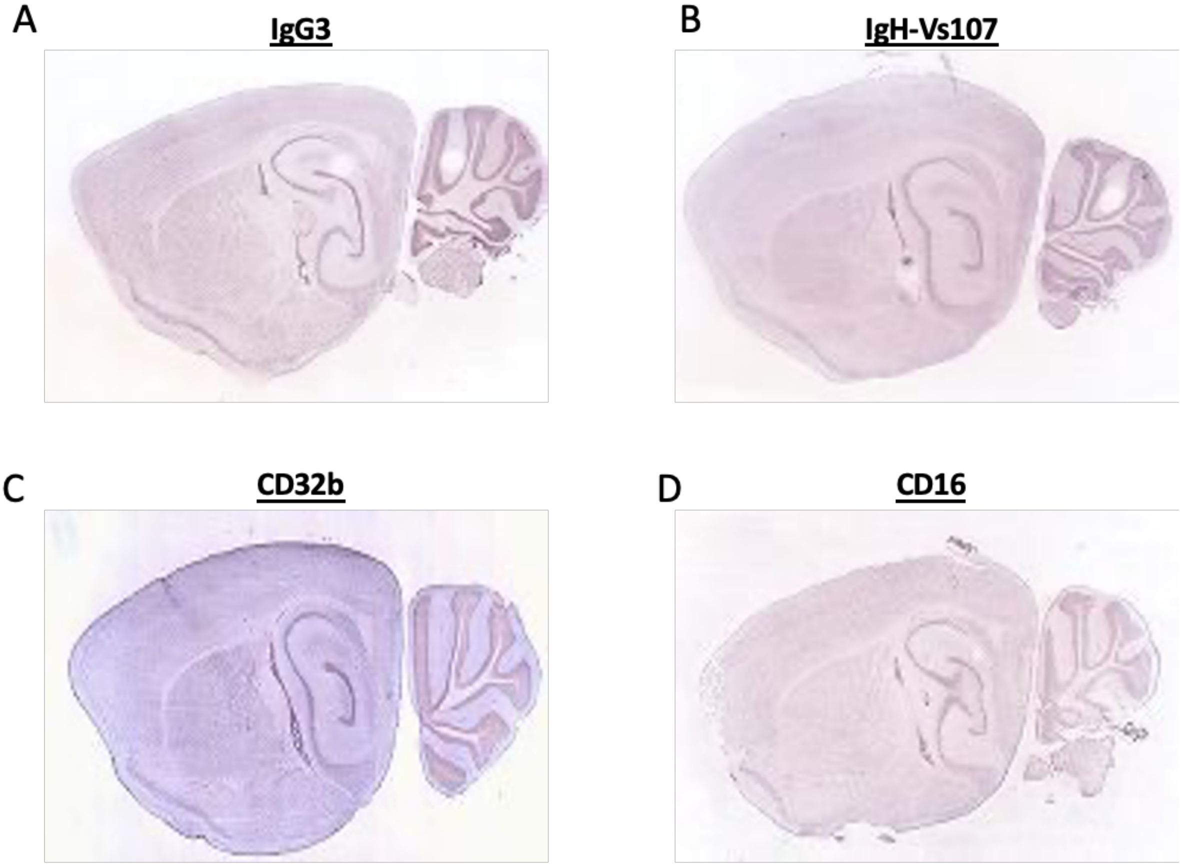

Supp. Figure 1: **A)** *IgG3*, **B)** *IgH-Vs107*, **C)** *Cd32b* and **D)** *CdD16* mRNA expression in Hippocampus and in Cerebellum of mice according to Allen Brain atlas, mRNA expression.
