# Supplementary material for: The Antibody Dependant Neurite Outgrowth Modulation Response Involvement in Spinal Cord Injury
Source: Front Immunol. 2022 Jun 16;13:882830. doi: 10.3389/fimmu.2022.882830 (PMC9245426; doi:10.3389/fimmu.2022.882830)
Supplement: Supplementary Figure 2 — (A) IgG3 mRNA expression in the spinal cord; (B) of mice according to the Allen Brain Atlas, mRNA expression. [file Presentation_2.pdf]

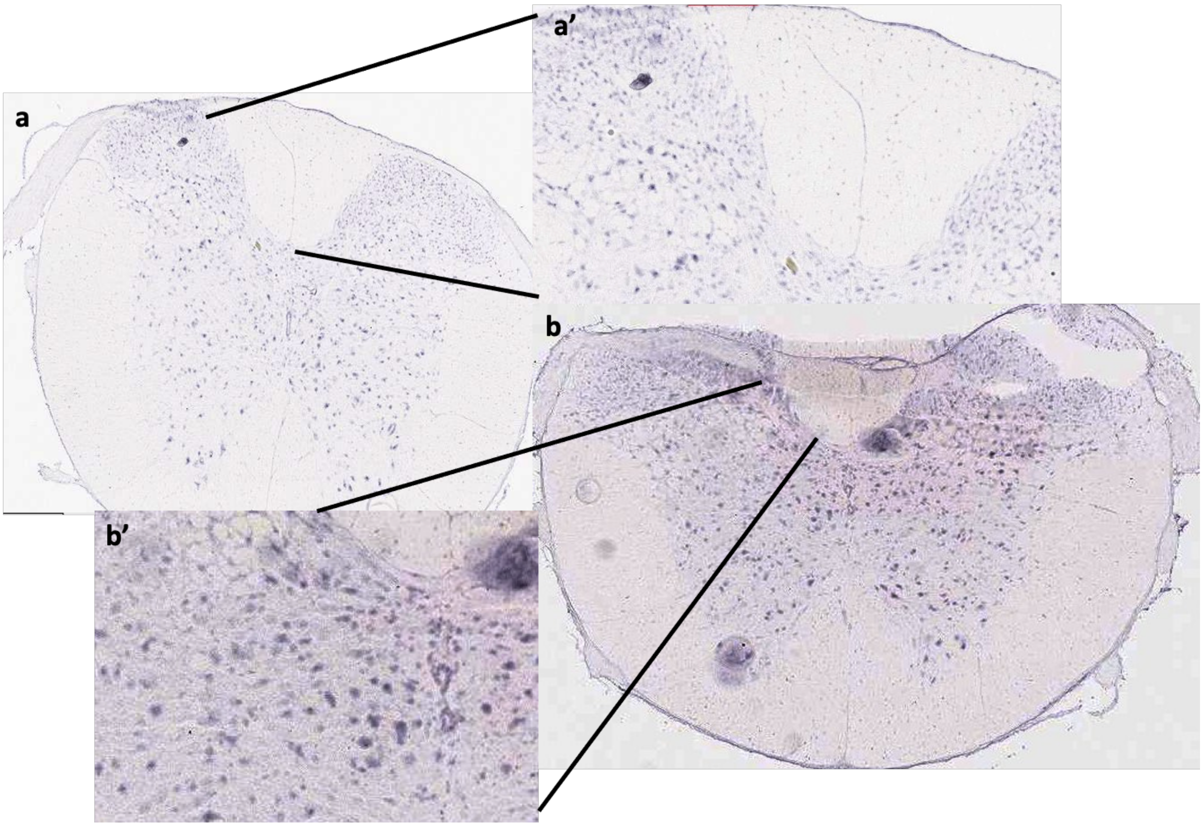

Supp. Figure 2: A) *IgG3* mRNA expression in spinal cord; of mice according to Allen Brain atlas, mRNA expression.
