# Supplementary material for: The Antibody Dependant Neurite Outgrowth Modulation Response Involvement in Spinal Cord Injury
Source: Front Immunol. 2022 Jun 16;13:882830. doi: 10.3389/fimmu.2022.882830 (PMC9245426; doi:10.3389/fimmu.2022.882830)
Supplement: Supplementary Figure 3 — Heatmap illustrating hierarchical clustering obtained after analysis of digested peptides isolated from rostral, caudal, and lesion segments collected 12 h after lesion and treatment with RhoA inhibitor. Cluster 1 corresponds to the inflammatory proteins and immunoglobulins. Cluster 2 contains proteins involved in synaptogenesis, and Cluster 3 proteins are involved in neurite outgrowth. [file Presentation_3.pdf]

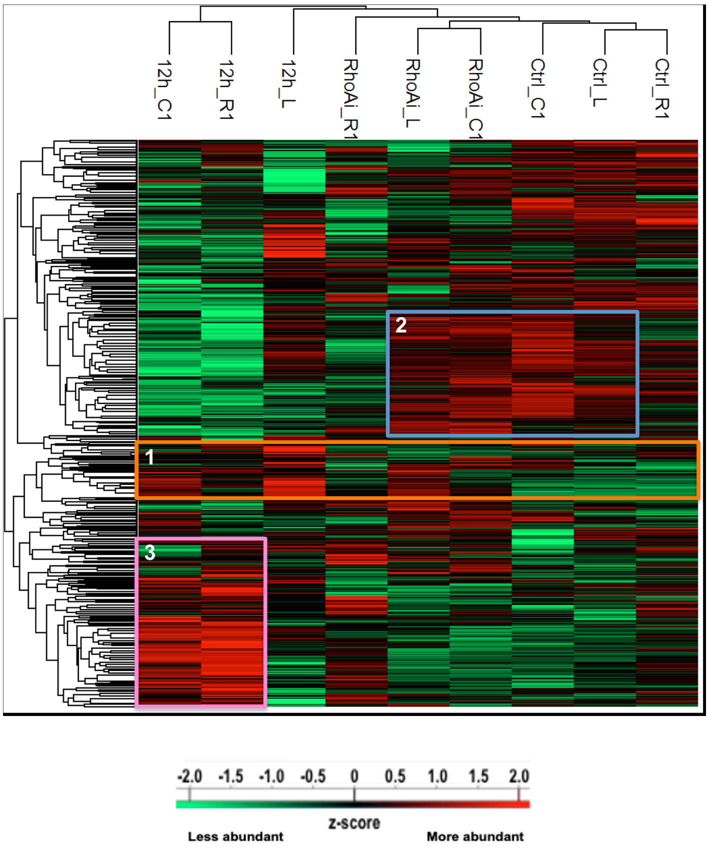

Supp. Figure 3: Heatmap illustrating hierarchical clustering obtained after analysis of digested peptides isolated from rostral, caudal and lesion segments collected 12 h after lesion and treatment or not with RhoA inhibitor. Cluster 1 corresponds to the inflammatory proteins and immunoglobulins. Cluster 2 contains proteins involved in synaptogenesis and Cluster 3, proteins involved in neurite outgrowth.
