# Supplementary material for: The Antibody Dependant Neurite Outgrowth Modulation Response Involvement in Spinal Cord Injury
Source: Front Immunol. 2022 Jun 16;13:882830. doi: 10.3389/fimmu.2022.882830 (PMC9245426; doi:10.3389/fimmu.2022.882830)
Supplement: Supplementary Figure 4 — Rodent mRNA species after NGS sequencing showing the highest fold changes in EAE spinal astrocytes as compared to control spinal cord astrocytes IgHa, Ig Kappa c, J Chain, and Cd32b mRNA have been identified in astrocytes from the spinal cord and the cortex. [file Presentation_4.pdf]

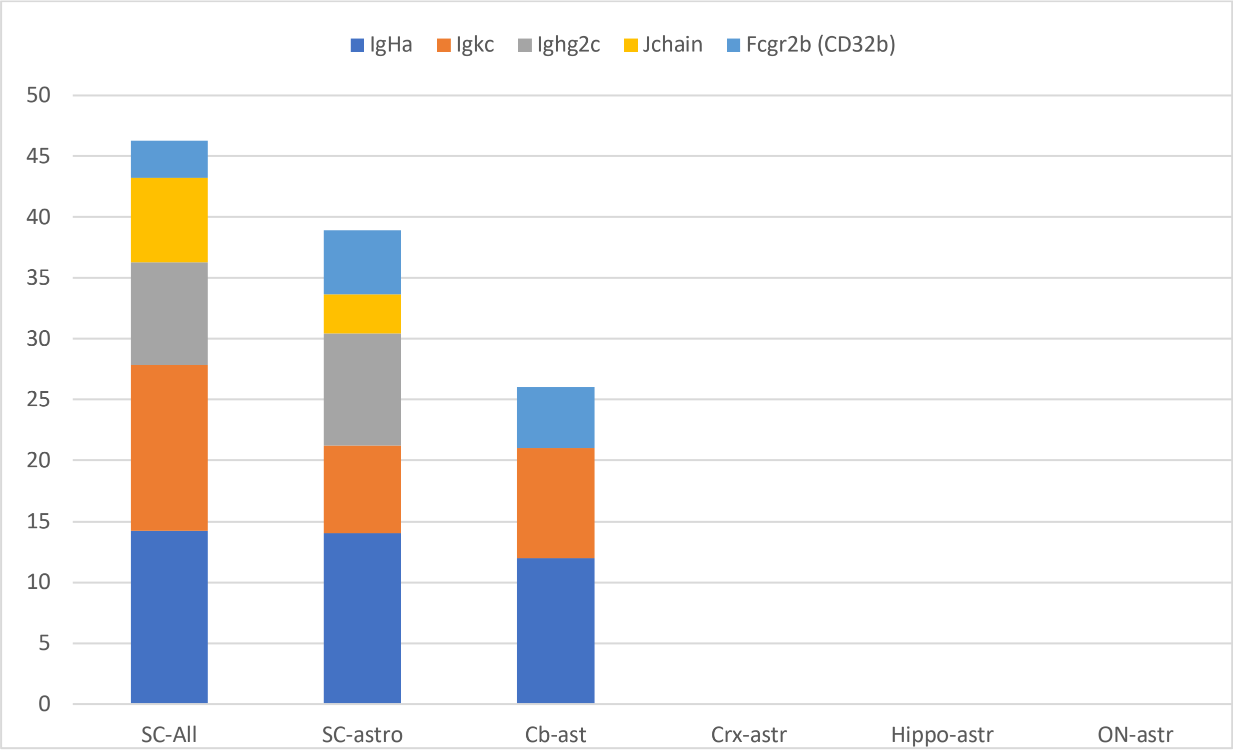

Supp. Figure 4: Rodent mRNA species after NGS sequencing showing the highest fold changes in EAE spinal astrocytes as compared to control spinal cord astrocytes *IgHa*, *Ig Kappa c*, *J Chain* and *Cd32b* mRNA have been identified in astrocytes from spinal cord and Cortex.
