# Supplementary material for: The Antibody Dependant Neurite Outgrowth Modulation Response Involvement in Spinal Cord Injury
Source: Front Immunol. 2022 Jun 16;13:882830. doi: 10.3389/fimmu.2022.882830 (PMC9245426; doi:10.3389/fimmu.2022.882830)
Supplement: Supplementary file 10 [file CD19.pdf › 1. SRX11310972 Rat adulte DRG/CD19.pdf]

NCBI Multiple Sequence Alignment Viewer, Version 1.21.0

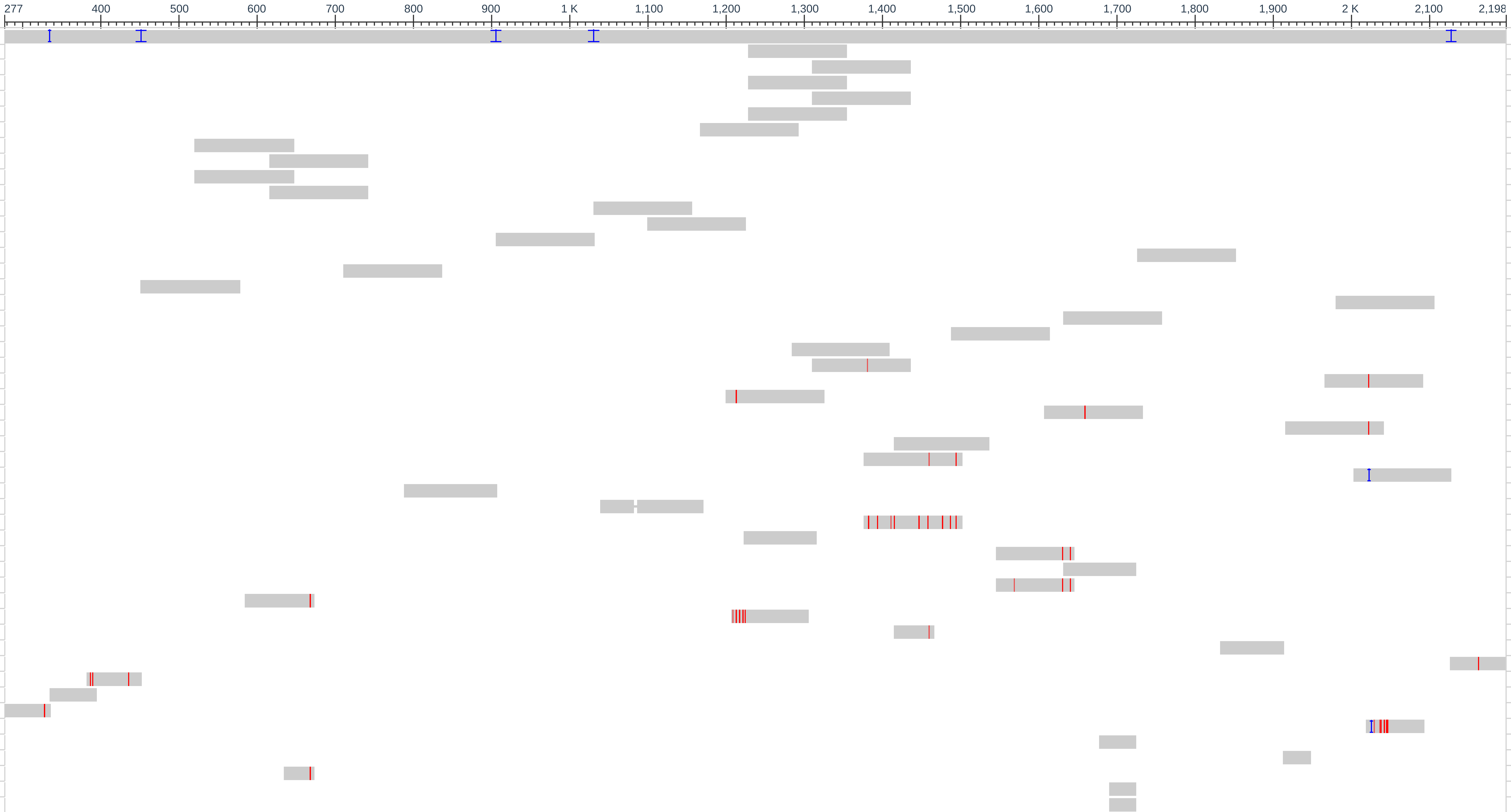

Sequence ID	Start	Alignment	End	Organism	Identity	Coverage	Mismatches
Query_27013 (+)	277		2,304		100.00	100.00	0
SRA:SRR149986..(+)	1		125		100.00	6.50	0
SRA:SRR149986..(-)	125		1		100.00	6.50	0
SRA:SRR149986..(+)	1		125		100.00	6.50	0
SRA:SRR149986..(-)	125		1		100.00	6.50	0
SRA:SRR149986..(+)	1		125		100.00	6.50	0
SRA:SRR149986..(+)	1		125		100.00	6.50	0
SRA:SRR149986..(+)	1		125		100.00	6.50	0
SRA:SRR149986..(-)	125		1		100.00	6.50	0
SRA:SRR149986..(+)	1		125		100.00	6.50	0
SRA:SRR149986..(-)	125		1		100.00	6.50	0
SRA:SRR149986..(+)	1		125		100.00	6.50	0
SRA:SRR149986..(-)	125		1		100.00	6.50	0
SRA:SRR149986..(+)	1		125		100.00	6.50	0
SRA:SRR149986..(-)	125		1		100.00	6.50	0
SRA:SRR149986..(+)	1		125		100.00	6.50	0
SRA:SRR149986..(-)	125		1		100.00	6.50	0
SRA:SRR149986..(-)	125		1		100.00	6.50	0
SRA:SRR149986..(-)	125		1		100.00	6.50	0
SRA:SRR149986..(+)	1		125		100.00	6.50	0
SRA:SRR149986..(-)	125		3		100.00	6.40	0
SRA:SRR149986..(-)	125		1		99.20	6.50	1
SRA:SRR149986..(-)	125		1		99.20	6.50	1
SRA:SRR149986..(-)	125		1		99.20	6.50	1
SRA:SRR149986..(+)	1		125		99.20	6.50	1
SRA:SRR149986..(+)	1		125		99.20	6.50	1
SRA:SRR149986..(-)	120		1		100.00	6.24	0
SRA:SRR149986..(+)	1		125		98.40	6.50	2
SRA:SRR149986..(-)	125		1		98.40	6.40	0
SRA:SRR149986..(-)	125		9		100.00	6.09	0
SRA:SRR149986..(-)	125		1		96.15	6.50	0
SRA:SRR149986..(+)	1		125		92.00	6.50	10
SRA:SRR149986..(+)	33		125		100.00	4.84	0
SRA:SRR149986..(-)	125		28		97.96	5.10	2
SRA:SRR149986..(+)	1		91		100.00	4.73	0
SRA:SRR149986..(-)	125		28		96.94	5.10	3
SRA:SRR149986..(+)	1		87		98.85	4.53	1
SRA:SRR149986..(+)	29		125		94.85	5.05	5
SRA:SRR149986..(+)	2		51		98.00	2.60	1
SRA:SRR149986..(+)	1		81		100.00	4.21	0
SRA:SRR149986..(+)	1		71		98.59	3.69	1
SRA:SRR149986..(+)	56		125		95.71	3.64	3
SRA:SRR149986..(-)	69		11		100.00	3.07	0
SRA:SRR149986..(+)	68		125		98.28	3.02	1
SRA:SRR149986..(-)	75		1		89.33	3.80	6
SRA:SRR149986..(-)	125		80		100.00	2.39	0
SRA:SRR149986..(+)	91		125		100.00	1.82	0
SRA:SRR149986..(-)	125		89		97.30	1.93	1
SRA:SRR149986..(+)	1		32		100.00	1.66	0
SRA:SRR149986..(+)	1		32		100.00	1.66	0
